# Cell-free DNA in precision medicine: overcoming biological barriers through integrated nanoparticle platforms for simultaneous diagnosis and therapy

**DOI:** 10.1186/s12951-026-04220-9

**Published:** 2026-02-27

**Authors:** Neda Esfandiari, Amirhossein Kaffash Arani, Armineh Jamshidi, Omid Miri, Sebastian P. Schwaminger

**Affiliations:** 1 NanoLab, Division of Medicinal Chemistry, Otto Loewi Research Center, Medical University of Graz, Graz, Austria; 2 Division of Physiology and Pathophysiology, Otto Loewi Research Center for Vascular Biology, Immunology and Inflammation, Medical University of Graz, Graz, Austria; 3 Department of Molecular Medicine and Genetics, School of Medicine, Hamadan University of Medical Sciences, Hamadan, Iran; 4 Department of Cellular and Molecular Biology, Faculty of Life Sciences and Biotechnology, Shahid Beheshti University, Tehran, Iran; 5 BioTechMed-Graz, Mozartgasse 12/II, Graz, 8010 Austria

**Keywords:** Cell-free DNA, Nanoparticles, Diagnostics, Therapy, Cancer, Fetal, Rheumatoid arteries, Sepsis, Precision medicine

## Abstract

Cell-free DNA (cfDNA), released into the bloodstream through cell death and cellular stress, has emerged as a highly promising noninvasive biomarker with broad diagnostic and therapeutic potential. However, clinical translation of cfDNA remains limited by its intrinsic properties, including extensive fragmentation, very low circulating concentrations, and extreme susceptibility to degradation by blood nucleases. Recent advances in nanoparticle-based nanotechnology have provided powerful solutions to overcome these barriers. In diagnostics, functionalized nanoparticles enable ultrasensitive and highly specific detection of cfDNA directly in blood, thereby facilitating early cancer detection, noninvasive prenatal screening, and real-time monitoring of diverse pathologies. Beyond diagnostics, cationic and multifunctional nanoparticles can actively bind and remove extracellular cfDNA from circulation, neutralizing its pro-inflammatory role as a damage-associated molecular pattern in conditions such as sepsis, rheumatoid arthritis, and cancers. Despite these advances, no comprehensive review has yet integrated cfDNA-based diagnostic and therapeutic strategies within a unified nanotechnological framework. Existing studies remain largely siloed, focusing independently on oncological biosensors, prenatal testing, or inflammatory processes in specific diseases. This fragmentation has hindered synergistic translational progress and efficient clinical implementation. This review systematically examines recent interdisciplinary advances at the intersection of nanotechnology and cfDNA research. By highlighting current challenges and opportunities, it outlines future directions toward realizing precision medicine applications that simultaneously exploit the diagnostic and therapeutic potential of cfDNA through integrated nanoparticle platforms.

**Graphical Abstract:**

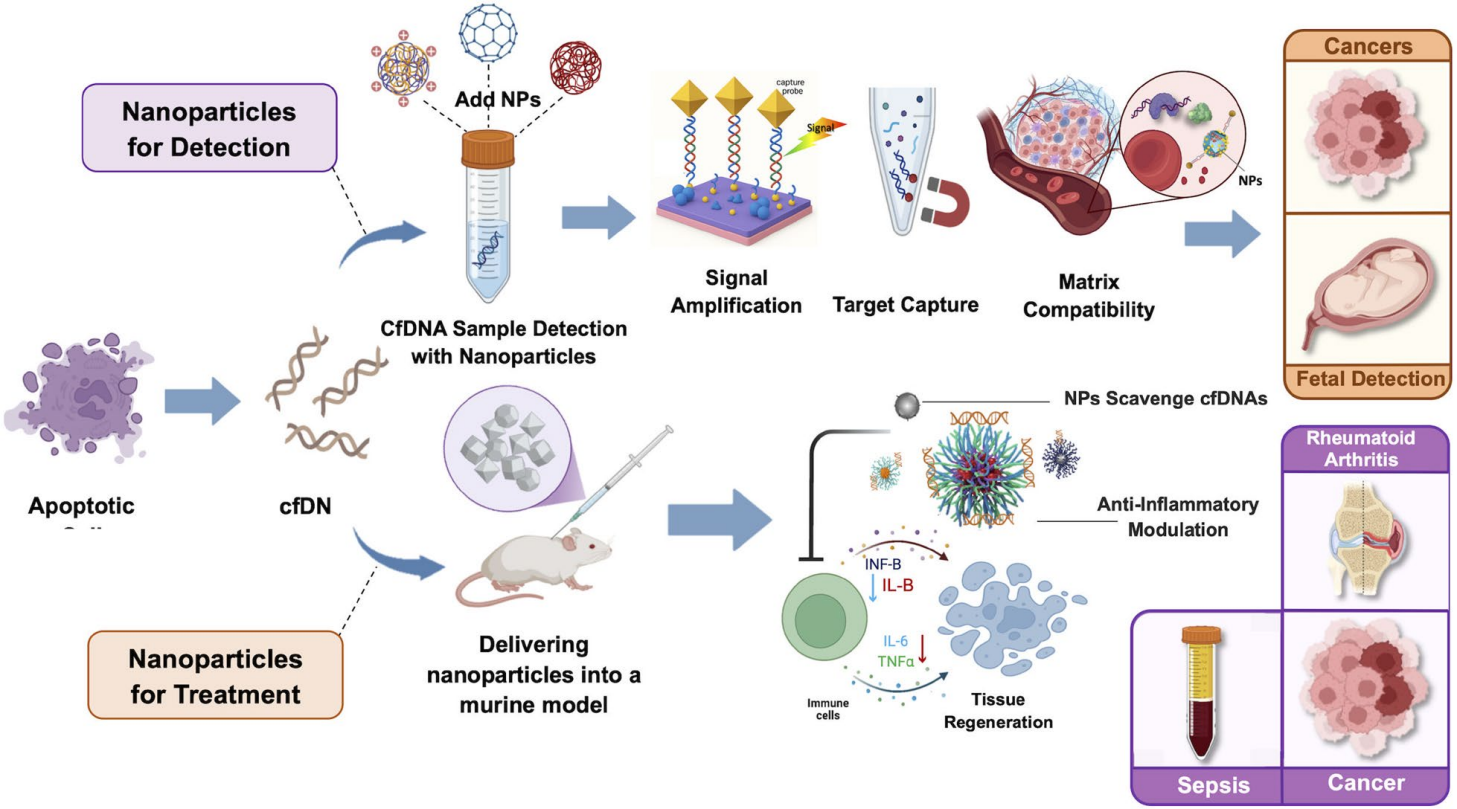

**Supplementary Information:**

The online version contains supplementary material available at 10.1186/s12951-026-04220-9.

## Introduction

The emergence of precision medicine has transformed disease diagnosis and treatment, with cell-free DNA (cfDNA) playing a central role as a powerful biomarker. cfDNA, fragmented DNA circulating in bodily fluids such as blood, urine, and cerebrospinal fluid, mainly originates from apoptotic or necrotic cells, but in cancer diagnostics, non-apoptotic and non-necrotic cells also contribute via active secretion, releasing nucleosomal fragments from nuclear DNA and nucleoid-like or intact mitochondrial DNA particles within extracellular vesicles [1–3]. Its clinical utility is well established, particularly in oncology for non-invasive cancer detection and monitoring, as well as in prenatal diagnostics for early screening of genetic abnormalities [4–6]. While diagnostic and therapeutic applications of cfDNA are often investigated separately, this review highlights the dual potential of nanoparticles in both domains, presenting them under a unified framework as complementary tools for advancing cfDNA-based precision medicine.

The increasing utilization of cfDNA-based testing highlights its capacity to advance personalized medicine by providing minimally invasive insights into disease states and treatment responses. In oncology, liquid biopsy techniques, particularly those analyzing cfDNA and circulating tumor DNA (ctDNA), are pivotal for cancer prognosis, genomic profiling, therapy selection, and monitoring treatment efficacy, despite challenges in standardization and clinical validation [7–9]. Similarly, cell-free fetal DNA (cffDNA) has transformed non-invasive prenatal testing (NIPT), enabling early detection of chromosomal abnormalities and monogenic disorders through advanced methods like next-generation sequencing, though confirmatory invasive tests are still required [10]. Beyond these, cfDNA shows significant potential in managing immune conditions like sepsis, a life-threatening disorder marked by an extreme immune response to infection, and rheumatoid arthritis (RA), a chronic autoimmune disease causing painful, swollen joints [11–14]. The remarkable versatility of cfDNA positions it as a powerful liquid biopsy tool, offering real-time, dynamic monitoring of disease progression and treatment response through minimally invasive sampling. Advances in nanobiotechnology have enabled the development of novel therapeutic strategies by elucidating the interplay between cfDNA and inflammatory pathways in disease pathogenesis [15].

Nanobiotechnology offers transformative potential in healthcare by enabling precise, molecular-level interventions that enhance therapeutic outcomes while reducing adverse effects [16–21]. The application of nanotechnology has significantly advanced the utility of cfDNA by overcoming challenges in its isolation, detection, and therapeutic delivery [22, 23]. Nanoparticles, with their high surface area-to-volume ratio and customizable properties, serve as essential tools in cfDNA-based diagnostics and treatments. Numerous studies highlight nanotechnological strategies for analyzing ctDNA, emphasizing the pivotal role of nanoparticles in diagnostic applications [24]. In the context of diagnostics, nanoparticles enable earlier and more accurate detection of diseases like cancer and fetal abnormalities by selectively binding small cfDNA fragments, amplifying detection signals, and reducing time-to-result compared to traditional method [24–26]. This diagnostic enhancement is underpinned by a triadic framework: (1) signal amplification, which boosts the sensitivity to detect low-abundance cfDNA through mechanisms like enzymatic or fluorescent enhancement, allowing for identification of subtle biomarkers; (2) target capture, which facilitates selective isolation of specific cfDNA sequences via surface-functionalized nanoparticles, improving specificity and reducing non-specific binding; and (3) matrix compatibility, which ensures robust performance in complex biological fluids by minimizing interference from proteins or other contaminants [4, 27, 28]. Each element of this framework independently contributes to overall diagnostic efficiency, enabling ultrasensitive and reliable cfDNA analysis.

Building on these diagnostic advances, therapeutic applications of nanoparticles primarily involve cationic or nucleic-acid-binding variants that directly bind extracellular cfDNA and neutralize its pro-inflammatory effects. For instance, in inflammatory conditions like rheumatoid arthritis or sepsis, where cfDNA promotes inflammation through immune activation, administering cfDNA-scavenging nanoparticles can reduce inflammation and improve clinical outcomes [29–33]. The integration of NPs with cfDNA technologies not only enhances diagnostic precision but also facilitates innovative treatment approaches by leveraging the unique physicochemical properties of nanomaterials.

A literature search was conducted in major databases including PubMed, Scopus, and Web of Science, focusing primarily on articles published between 2020 and 2026 (with limited inclusion of highly relevant earlier works). Search terms combined variations of “cell-free DNA/RNA” (e.g., cfDNA, ctDNA, circulating DNA, cell-free nucleic acids) with “nanoparticle” and related terms (e.g., nanocrystals, nanocrystalline materials). In total, approximately 420 records were identified, of which 110 met our inclusion criteria (peer-reviewed original research or review articles in English directly addressing nanoparticle applications in cfDNA/cfRNA isolation, detection, or therapeutic modulation) and were selected for this narrative review.

.

Emerging technologies, such as artificial intelligence (AI), further present opportunities to optimize detection algorithms, simulate therapeutic responses, and reduce errors and population biases in nanoparticle-assisted cfDNA applications [34]. By bridging these interdisciplinary areas, this review seeks to shed light on current trends, address challenges, and predict the future direction of nanobiotechnology in precision medicine.

## Nanoparticles and cfDNA in diagnosis

### Nanoparticle-enhanced detection of ctDNA

**Fig. 1:**
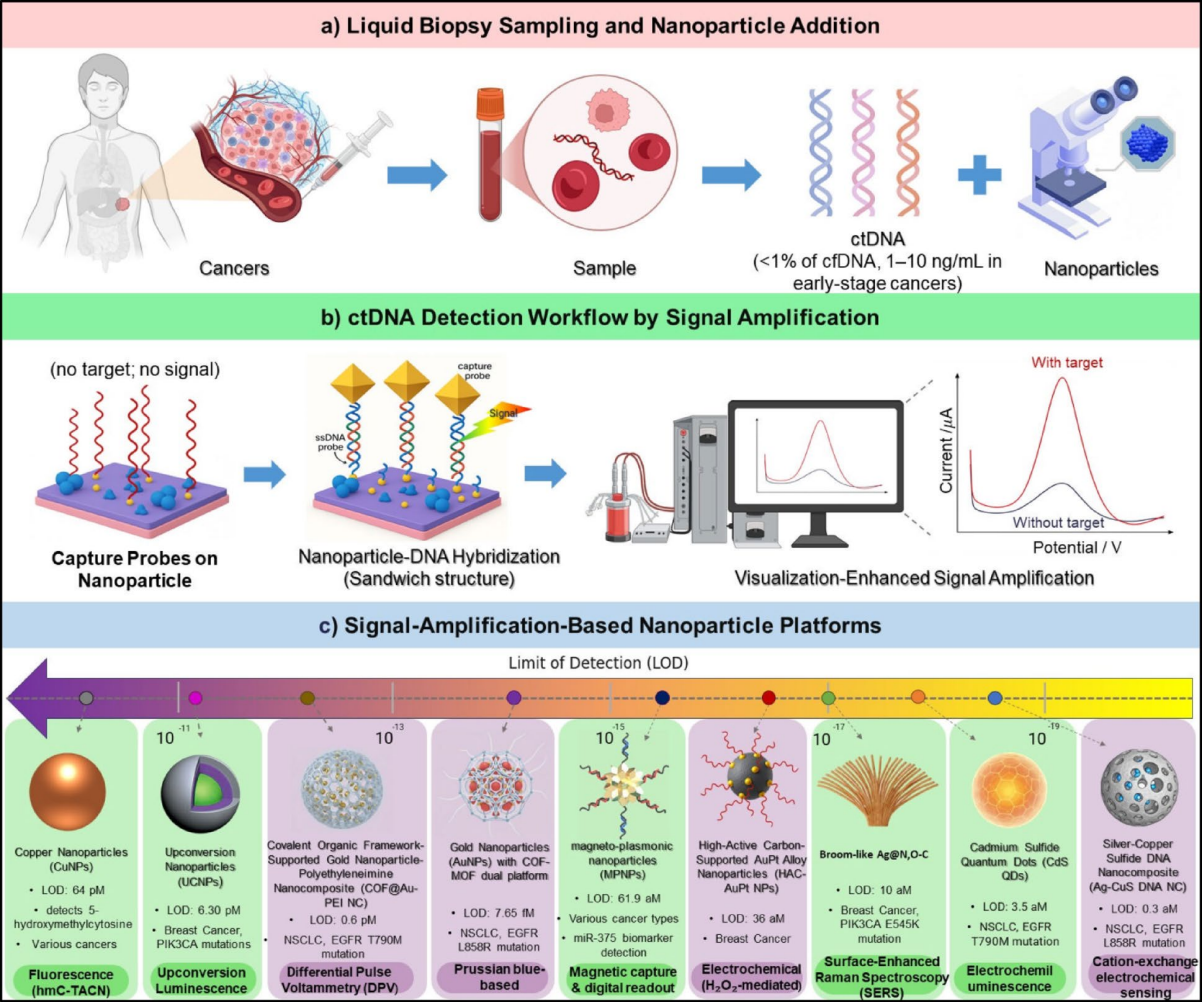
Nanoparticle-assisted ctDNA extraction and signal-amplified detection strategies. (**a**) Liquid biopsy sampling from cancer patients, where circulating tumor DNA (ctDNA), present at extremely low abundance and in short fragment sizes, is isolated from blood samples using nanoparticle-based approaches to enhance capture efficiency and signal strength. (**b**) Sandwich-type detection strategy, in which target ctDNA is simultaneously recognized by surface-immobilized capture probes and nanoparticle-conjugated detection probes through complementary base pairing. The formation of this sandwich architecture generates an initial detection signal and subsequently triggers cascade signal-amplification processes, which are finally recorded by the detector. (**c**) Signal-amplification–based nanoparticle platforms for ctDNA detection, categorized into electrochemical approaches (purple) and optical approaches (green). The platforms are arranged according to their reported limits of detection (LOD), with the corresponding cancer-related mutations identified for each sensing strategy. Part B of this figure is reprinted from Guo L, Mu Z, Yan B, Wang J, Zhou J, Bai L. A novel electrochemical biosensor for sensitive detection of non-small cell lung cancer ctDNA using NG-PEI-COFTAPB-TFPB as sensing platform and Fe-MOF for signal enhancement. Sensors and Actuators B: Chemical. 2022; 350:130874. 10.1016/j.snb.2021.130874., with permission from Elsevier. Created in BioRender. Schwaminger, S. (2026) https://BioRender.com/5y0aqdd

CtDNA, a subset of cfDNA released by tumor cells into biofluids such as plasma, serum, or urine, is a critical non-invasive biomarker for early cancer detection, personalized therapy, and disease monitoring [8, 35, 36]. Bearing tumor-specific genetic and epigenetic alterations, such as *EGFR* mutations in non-small cell lung cancer (NSCLC), including *EGFR* T790M and *EGFR* L858R, or *PIK3CA* mutations in breast cancer, ctDNA offers a comprehensive molecular profile, circumventing the invasiveness and limited tumor representation of tissue biopsies [8, 35]. The L858R mutation is an activating alteration that enhances EGFR signaling and confers sensitivity to first-generation tyrosine kinase inhibitors (TKIs), whereas the T790M mutation represents a secondary resistance mechanism that diminishes TKI efficacy and often emerges after initial therapy, thereby necessitating the use of next-generation inhibitors [37]. Similarly, *PIK3CA* mutations, commonly occurring in hormone receptor–positive breast cancer, lead to constitutive activation of the PI3K/AKT/mTOR pathway, promoting tumor growth and therapeutic resistance [38]. Despite its potential, ctDNA detection is hindered by its low abundance (< 1% of cfDNA, 1 to 10 ng/mL in early-stage cancers), short fragment length (< 200 bp), and presence in complex biofluids containing proteins and nucleases, necessitating diagnostic platforms with exceptional sensitivity and specificity (Fig. 1a) [39–41]. Conventional methods like quantitative PCR and next-generation sequencing are limited by high costs, prolonged processing times, and insufficient sensitivity for trace ctDNA levels [41, 42].

NPs, leveraging properties such as localized surface plasmon resonance (LSPR), superparamagnetism, and high surface-to-volume ratios, provide a solution through a triadic framework of signal amplification, target capture, and matrix compatibility. Advanced methodologies are critical for achieving ultrasensitive, specific, and reliable detection of circulating tumor DNA (ctDNA), facilitating significant clinical progress in the management of cancers such as NSCLC, breast cancer, hepatocellular carcinoma (HCC), colorectal cancer (CRC), prostate cancer, pancreatic cancer, and glioma [43–48].

#### Signal amplification

Nanoparticles play a vital role in detecting ctDNA because they can amplify signals to overcome the challenge of ctDNA’s low abundance in blood or other samples. This signal amplification, a core part of the triadic framework, allows detection at incredibly low concentrations, from attomolar (aM) to femtomolar (fM) levels. Gold nanoparticles (AuNPs) are especially important here, as they use LSPR to boost both electrochemical and optical signals [4]. A powerful approach to achieve this high sensitivity is the sandwich-type hybridization format. In this setup, short DNA sequences called capture probes are attached to conductive surfaces like gold electrodes or covalent organic frameworks (COFs). At the same time, signal probes are linked to nanostructures such as AuNPs, metal-organic frameworks (MOFs), or hybrid nanohybrids. When target ctDNA is present, it binds to both probes, forming a stable sandwich complex that positions the nanostructures close to the sensing surface. This arrangement concentrates catalytic or optical components, leading to strong signals through electrochemical reactions, such as hydrogen peroxide (H₂O₂) reduction or the formation of Prussian blue (a deep-blue compound created when ferricyanide and ferrous ions react, producing a measurable current), or through optical changes like shifts in LSPR. This design reliably detects ctDNA at aM to fM levels with the ability to spot single-base differences, making it the foundation for many cutting-edge biosensors (Fig. 1b) [39, 49, 50].

To make the amplification strategies clearer, we can group them into electrochemical and optical methods. These approaches work together seamlessly, using shared nanostructures and the sandwich hybridization format to detect ctDNA with remarkable precision and sensitivity.

##### Electrochemical approaches

Electrochemical methods use nanoparticles to boost electron transfer or trigger catalytic reactions, creating strong, measurable current signals. For example, a biosensor developed for the *EGFR* L858R mutation in non-small cell lung cancer (NSCLC) combined two nanostructures to enhance performance [39]. A COF, blended with nitrogen-doped graphene and polyethyleneimine, created a highly conductive surface with plenty of binding sites for capture DNA probes, thanks to strong interactions between gold and nitrogen. Meanwhile, an iron-based MOF decorated with AuNPs carried signal probes and amplified the signal by producing Prussian blue, which generates a distinct current. This system could detect ctDNA at a limit of 7.65 fM, with a detection range from 100 fM to 100 nM. It also showed excellent reproducibility, with a relative standard deviation (RSD) of about 1.3%, and recovered 90 to 102% of ctDNA in serum samples, proving its potential for early NSCLC diagnosis [39]. Another electrochemical biosensor focused on the *EGFR* T790M mutation in NSCLC, using a technique called differential pulse voltammetry (DPV) [51]. This sensor used a COF coated with 17-nm AuNPs modified with polyethyleneimine, which improved electron transfer and securely anchored hairpin DNA probes specific to T790M ctDNA. When the target ctDNA bound, Au@Pt nanohybrids acting as signal amplifiers reduced the DPV current, allowing detection at 0.6 pM with 95.5% accuracy and an RSD of 1.2% across 30 samples. Its ability to distinguish single-base mismatches made it valuable for guiding treatment decisions [51].

A highly sensitive electrochemical biosensor for *EGFR* L858R detection took a different approach, using a dual-amplification strategy [46]. It relied on DNA nanospheres that trapped silver ions (Ag⁺) and CuS nanoparticles for cation exchange. When ctDNA bound, the nanospheres broke apart, releasing Ag⁺ ions that prompted Cu²⁺ release from CuS nanoparticles, boosting the electrochemical signal. This method achieved an extraordinary detection limit of 0.3 aM, with a range from 1 aM to 1 fM, and recovered 95 to 105% of ctDNA in 42 clinical samples. Its results matched well with qPCR, CT scans, and pathology reports, showing its promise for fast and accurate NSCLC diagnosis [46]. For breast cancer, an electrochemical biosensor used high-active carbon-supported AuPt alloy nanoparticles (HAC-AuPt) to detect ctDNA [49]. Capture probes on gold electrodes bound the target, while HAC-AuPt-labeled signal probes triggered H₂O₂ reduction, producing a strong current. This setup detected ctDNA at 36 aM, with a range from 10⁻⁸ to 10⁻¹⁶ M, an R² of 0.994, and 92.6 to 105.1% recovery with less than 5% RSD in 10 serum samples, offering great precision for early diagnosis [49]. These electrochemical methods all build on nanoparticle-driven reactions and the sandwich format to deliver highly sensitive ctDNA detection.

##### Optical approaches

Optical methods, often utilizing nanostructures shared with electrochemical systems, employ light-based signals such as LSPR, Surface-Enhanced Raman Spectroscopy (SERS), fluorescence, or electrochemiluminescence for ctDNA detection. for example, a breast cancer study used silver nanoparticles (AgNPs) with a broom-like morphology, coated with nitrogen and oxygen-doped carbon, for SERS-based detection of *PIK3CA* E545K mutation ctDNA [52]. SERS allows for high resolution analysis of inelastic laser light scattering indicating material composition by a specific vibrational spectrum [41, 53]. The weak Raman effect is amplified by AgNPs through LSPR-induced electromagnetic fields and chemical interactions, increasing intensity by orders of magnitude. The broom-like AgNP structure maximized surface area, enhancing ctDNA interaction and SERS signal strength. This enabled detection at 10 aM, with an R² of 0.994 and 97.5% stability over 14 days in 22 plasma samples, offering a sensitive, non-invasive approach for monitoring breast cancer mutations [52].

In an NSCLC study, cadmium sulfide quantum dots (CdS QDs) were used in an electrochemiluminescence assay to detect *EGFR* T790M mutation ctDNA [54]. These tiny particles emit light when electrically stimulated, with the intensity depending on their size. When ctDNA bound to probes on a CdS QD-coated electrode, it triggered light emission, detecting ctDNA at 3.5 aM with an RSD of 2.6% in 15 serum samples. This approach is particularly good at detecting short ctDNA fragments, which traditional PCR often misses, improving early diagnosis [54].

A fluorescence-based assay called hmC TACN used copper (Cu) nanoparticles to detect 5-hydroxymethylcytosine (5hmC), an epigenetic DNA modification strongly associated with cancer. When ctDNA containing 5hmC binds to the TACN probe, Cu nanoparticles enhance the fluorescence intensity, resulting in a highly sensitive signal. Using this strategy, ctDNA with 5hmC was detected at concentrations as low as 64 pM in 22 clinical samples, demonstrating its potential for multi cancer detection [55].

Upconversion nanoparticles (UCNPs), doped with rare earth ions, absorb near-infrared light and emit visible or ultraviolet light, avoiding unwanted background light from biological samples. In breast cancer, UCNPs detected *PIK3CA* mutations at 6.30 pM with single-base specificity in 18 clinical samples, helping with precise mutation profiling for targeted treatments. These optical methods, often sharing nanostructures like AuNPs with electrochemical systems, use the sandwich format to concentrate light-based signals for high sensitivity [56].

A magnetic-activated capture and digital counting (mAC + DC) platform has been developed for rapid, enzyme-free, and ultrasensitive quantification of circulating microRNAs. The system employs spiky Fe₃O₄@Au magneto-plasmonic nanoparticles (MPNPs) functionalized with target-specific probes that hybridize with miRNA through toehold-mediated strand displacement. Upon binding, the activated MPNPs are magnetically guided toward complementary capture probes on a photonic crystal surface, overcoming diffusion limitations and enabling single-molecule capture. Spectral coupling between the LSPR of the MPNPs and the photonic crystal resonance allows each bound nanoparticle to be digitally visualized through local reflection quenching. This approach enabled direct detection of the miR-375 biomarker from unprocessed serum within one minute, achieving a 61.9 aM detection limit, wide dynamic range (100 aM–10 pM), and single-base mismatch selectivity, demonstrating strong potential for point-of-care cancer diagnostics [57].

These signal amplification strategies are indispensable, enabling detection of ctDNA at concentrations unattainable by conventional methods, thus facilitating early diagnosis and targeted therapy (Fig. 1c).

#### Target capture

The target capture approach is a key step in liquid biopsy that focuses on isolating and enriching small fragments of DNA circulating in the bloodstream. Its main goal is to selectively “fish out” tumor-derived genetic material from among many other components in blood or plasma. By using materials that can specifically recognize and bind to target DNA, often assisted by nanotechnology or magnetic particles, this method ensures that the captured DNA is pure and concentrated enough for precise molecular testing. Magnetic nanoparticles (MNPs), such as Fe₃O₄-based particles, leverage their superparamagnetic nature, magnetizing only when a field is applied and losing it afterward, to simplify isolation from complex fluids like plasma [58–62].

A microfluidic system integrating superparamagnetic silica-coated nanoparticles (Fe_3_O_4_@SiO_2_) for ctDNA isolation from plasma was introduced by Balakrishnan et al. [27]. The mechanism of capture in this design is based primarily on physical adsorption and electrostatic interactions between the negatively charged DNA backbone and the silica surface under chaotropic conditions. Plasma first flows through a size-selective filtration region that excludes larger particles, after which ctDNA fragments (~ 2.6 nm) interact with silica-coated magnetic beads in curved microchannels. The superparamagnetic behavior of the beads allows reversible magnetic trapping under a mild magnetic field (~ 10 mT), enabling DNA recovery without centrifugation. This approach yielded an average of 5.7 ng ctDNA from 10 mL plasma, with 65.57% sensitivity and 95.38% specificity in early-stage cancer samples, demonstrating the efficiency of magnetically assisted physical adsorption for cfDNA enrichment in a microfluidic environment [27].

The concept of chemical target capture was advanced through the development of an electrochemical nanobiosensor for detecting methylated cfDNA from the E-cadherin (*CDH1*) promoter region, a key tumor suppressor gene frequently silenced in epithelial cancers [63]. The magnetic nanoparticles (Fe₃O₄–citric acid, ~ 12 nm) in this system serve both as magnetic carriers and as reactive scaffolds. Their surface carboxyl groups, derived from citric acid, allow covalent immobilization of amino-modified single-stranded DNA probes via EDC/NHS coupling. These probes specifically hybridize with methylated CpG sites on cfDNA, while nonmethylated DNA remains unbound. After magnetic separation, the resulting complexes are transferred onto a reduced graphene oxide/polyvinyl alcohol electrode, where methylation-specific antibody binding is monitored by differential pulse voltammetry (DPV). This hybrid mechanism (combining covalent probe anchoring, sequence-specific hybridization, and electrochemical readout) enabled a detection limit of 9 × 10⁻⁵ ng/mL, showcasing the potential of magnetic nanoparticles in epigenetic cfDNA sensing with both chemical and electrical precision [63].

A highly refined size-selective magnetic capture system was developed for cfDNA extraction and ctDNA enrichment. The platform employs Fe₃O₄@SiO₂ core–shell nanoparticles (~ 500 nm), in which the Fe₃O₄ core enables rapid magnetic isolation, while the hydroxyl-rich silica shell captures DNA fragments via hydrogen bonding and electrostatic interactions in the presence of chaotropic salts and alcohols [64]. The capture mechanism relying on differences in fragment size and ionic strength. Under optimized conditions, long genomic DNA (> 550 bp) is first excluded under weak precipitant conditions, followed by selective adsorption of short cfDNA fragments (120 to 200 bp) representing ctDNA under stronger conditions. By fine-tuning the buffer ratio (*r* = 1.35), the system achieved > 90% recovery of short DNA fragments with < 0.1% contamination from long DNA. Subsequent NGS analysis showed nearly doubled mutation detection rates (*BRCA2*: 1.35→2.61%; *IDH1*: 1.58→3.46%) and revealed new *MSH6* variants associated with DNA repair defects. This two-step, size-dependent target capture highlights the power of controlled physicochemical parameters in achieving precise cfDNA separation and quantification [64].

Hybrid magnetic–plasmonic strategies have emerged as powerful approaches for sequence-specific cfDNA and ctDNA capture from plasma, employing 1 μm superparamagnetic microbeads functionalized with biotinylated oligonucleotides to enable magnetic isolation followed by plasmonic readout using single-base–discriminating PNA probes [65]. This capture-first strategy eliminates conventional cfDNA extraction and PCR amplification, enabling the selective enrichment and direct detection of clinically relevant KRAS single-nucleotide variants (SNVs), including the hotspot p.G13D mutation, from only a few microliters of plasma. KRAS SNVs, which are among the most prevalent oncogenic alterations in colorectal, pancreatic, and lung cancers, disrupt GTPase activity and drive constitutive signaling associated with poor prognosis and therapeutic resistance. Dyna@MIX, commercially available superparamagnetic microbeads, offer a high oligonucleotide loading density (≈ 3–4 × 10⁵ probes per bead), excellent colloidal stability, and minimal nonspecific adsorption, while their high refractive index (~ 1.6) significantly enhances optical mass loading and signal transduction in surface plasmon resonance imaging (SPRI) measurements. By employing dithiobis-succinimidyl-propionate (DTSP)-anchored peptide nucleic acid (PNA) probes (PNA-WT for the wild-type sequence and PNA-G13D for the mutant) immobilized at an ultrahigh density of ~ 9 × 10¹² molecules cm⁻², the system achieves an exceptionally low detection limit of 0.5 aM (≈ 300 copies mL⁻¹) for 1.0 pg µL⁻¹ spiked mutant DNA, as validated using colorectal cancer patient samples. DTSP serves as a bifunctional crosslinker that covalently anchors the PNA probes to the sensor surface via stable thiol–amine chemistry, ensuring robust probe orientation, high surface density, and minimal nonspecific adsorption. By integrating magnetic pre-concentration, optical signal amplification, and the inherent single-nucleotide hybridization specificity of PNA, this platform enables rapid, real-time, label-free detection of clinically relevant SNVs, providing an ultrasensitive and cost-effective approach for liquid biopsy analysis [65].

**Fig. 2:**
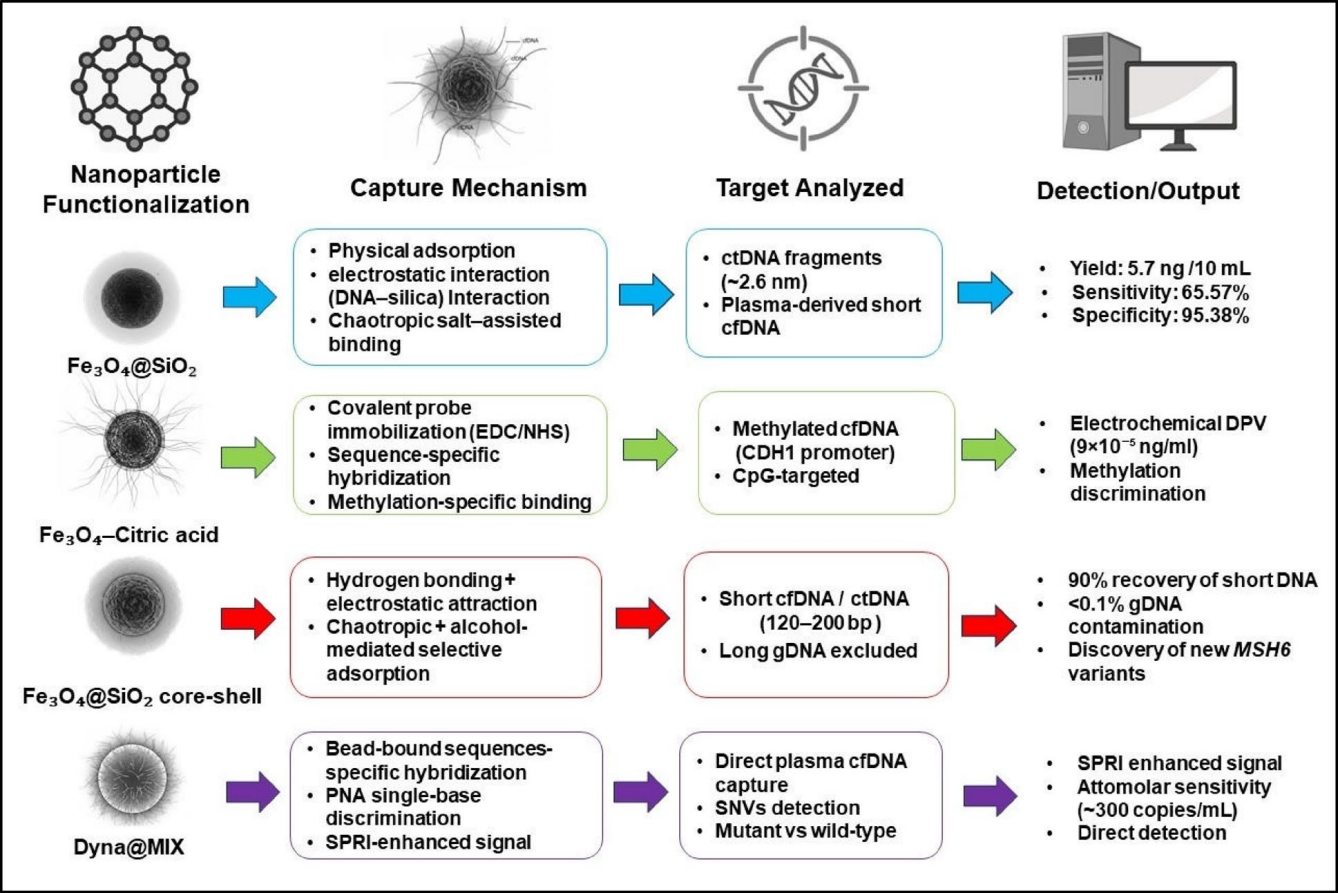
Magnetic target capture strategies for cfDNA/ctDNA. Fe₃O₄@SiO₂ nanoparticles use chaotropic salt-assisted physical adsorption and electrostatic interaction to isolate short plasma-derived ctDNA fragments (~ 2.6 nm) with a yield of 5.7 ng from 10 mL plasma, achieving 65.57% sensitivity and 95.38% specificity. Fe₃O₄–citric acid nanoparticles functionalized with covalently immobilized EDC/NHS-activated probes specifically bind CDH1 promoter–methylated cfDNA, enabling differential pulse voltammetry (DPV) detection with an LOD of 9 × 10⁻⁵ ng/mL. Fe₃O₄@SiO₂ core–shell nanoparticles enable hydrogen-bonding and chaotropic/alcohol-tuned selective adsorption to enrich short cfDNA/ctDNA (120–200 bp) while excluding long genomic DNA, achieving > 90% short-DNA recovery and < 0.1% gDNA contamination and enabling identification of new MSH6 variants. Dyna@MIX superparamagnetic microbeads facilitate direct hybridization in plasma, magnetically assisted separation, and SPRI-based plasmonic enhancement for distinguishing mutant from wild-type cfDNA and detecting single-nucleotide variants with attomolar sensitivity (~ 300 copies/mL) without PCR, extraction, or lengthy processing, all within < 2 h. Created in BioRender. Schwaminger, S. (2026) https://BioRender.com/plhzx5a

Taken together, these studies outline a clear technological continuum in magnetic nanomaterial–based cfDNA and ctDNA isolation. The capture mechanisms evolve from simple electrostatic adsorption and covalent probe hybridization to complex multi-modal processes integrating size-selective binding, covalent chemistry, and optical amplification. Each system leverages the magnetic control and nanoscale engineering of its materials to refine the target capture process, ultimately enhancing the accuracy, sensitivity, and clinical applicability of liquid biopsy–based cancer diagnostics (Fig. 2).

#### Matrix compatibility

**Fig. 3:**
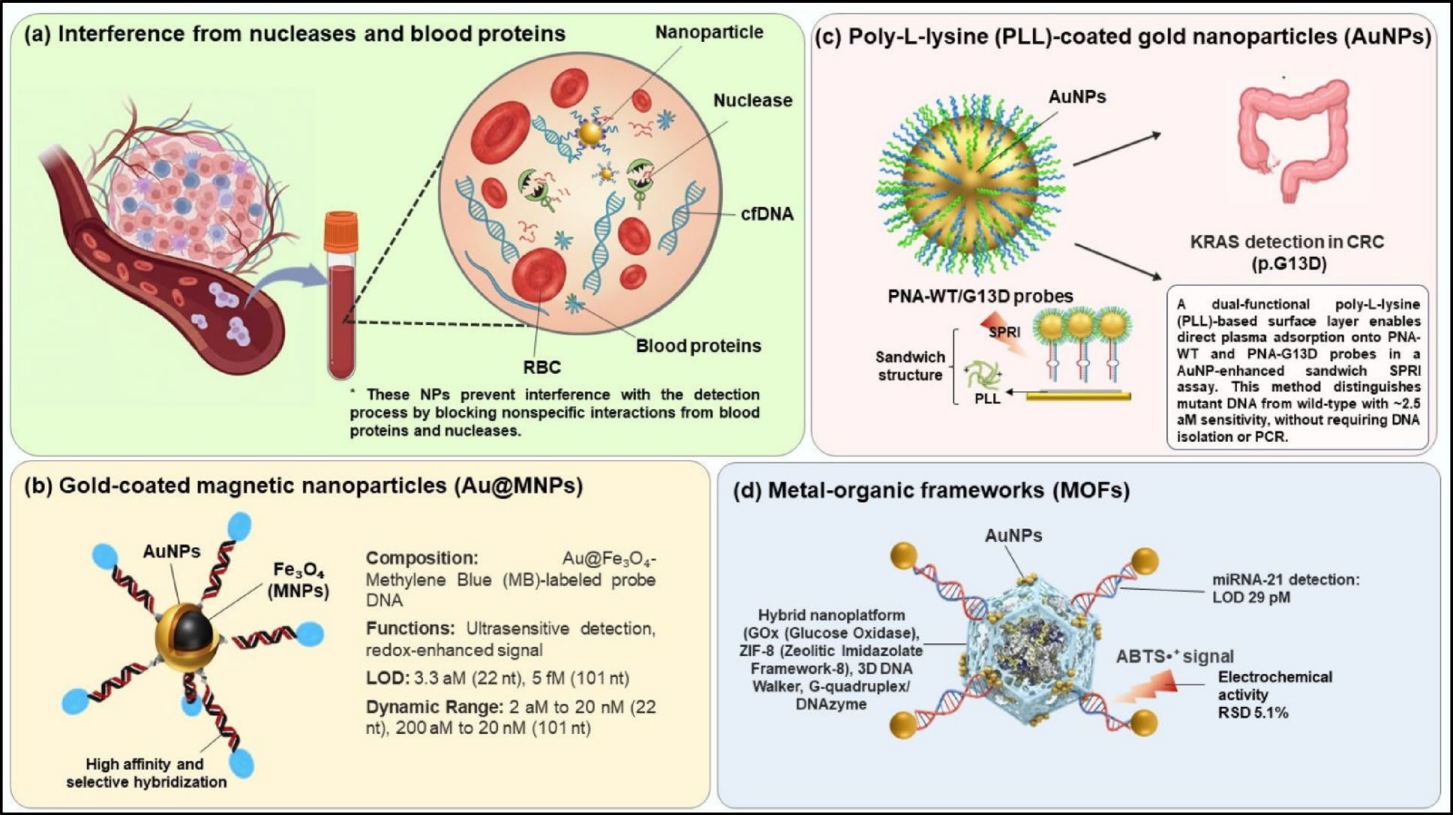
Matrix-compatible nanoparticle platforms for ultrasensitive ctDNA detection. Matrix-compatible nanoparticle platforms for ultrasensitive and interference-resistant detection of ctDNA in complex biofluids (**a**) Matrix compatibility reduces the impact of nucleases and abundant blood proteins that impair hybridization efficiency. (**b**) Gold-coated magnetic nanoparticles (Au@MNPs) paired with methylene blue–tagged DNA probes detect ctDNA through hybridization-disrupted electron tunneling and redox signal amplification, providing LODs of 3.3 aM (22 nt; 2 aM–20 nM range) and 5 fM (101 nt; 200 aM–20 nM) within 20 min. (**c**) Poly-L-lysine (PLL)-coated gold nanoparticles suppress nonspecific adsorption and detect KRAS p.G13D mutations at 2.5 aM directly in colorectal cancer plasma without PCR amplification. (**d**) Metal-organic frameworks (MOFs) integrated with gold nanoparticles and glucose oxidase (GOx) detect serum miRNA-21 at 29 pM; the MOF structure promotes efficient hybridization and catalysis, generating a 5.1% ABTS⁺ electrochemical signal via enzymatic coupling. Part of panel A of this figure was adapted from Omar, M. A., Omran, M. M., Farid, K., Tabll, A. A., Shahein, Y. E., et al. Biomarkers for hepatocellular carcinoma: from origin to clinical diagnosis. Biomedicines, 2022;11(7), 1852. 10.3390/biomedicines11071852. with permission from Elsevier. Created in BioRender. Schwaminger, S. (2026) https://BioRender.com/tvww9u2

Matrix compatibility in nanoparticle (NP)-based sensing systems is a critical requirement for reliable ctDNA detection in complex biofluids such as blood plasma, where nucleases, plasma proteins, and cellular components can severely compromise analytical sensitivity, specificity, and signal fidelity. As illustrated in Fig. 3a, matrix-compatible NPs mitigate these interferences by suppressing nonspecific interactions, including cfDNA degradation by nucleases and adsorption of blood proteins or red blood cells (RBCs), thereby reducing background noise, signal distortion, and false-positive responses arising from the biological matrix (Fig. 3a).

Gold-coated magnetic nanoparticles (Au@MNPs) represent a representative strategy in which matrix compatibility is achieved through physical separation and enrichment. These hybrid nanostructures consist of a magnetic Fe₃O₄ core and a gold shell functionalized with methylene blue (MB)-labeled probe DNA complementary to target ctDNA. During detection, the magnetic core enables efficient capture, concentration, and isolation of ctDNA directly from plasma or whole blood under an external magnetic field, eliminating the need for prior nucleic acid extraction. The MB moieties act as electrochemical reporters and undergo continuous redox cycling with ferricyanide, where methylene blue is reduced to leucomethylene blue (LB) and subsequently reoxidized, generating an amplified current proportional to the amount of hybridized ctDNA. Upon target binding, formation of a double-stranded DNA layer partially blocks electron tunneling between MB and the electrode, resulting in measurable current changes. This system enables detection of 22-nucleotide DNA from 2 aM to 20 nM (LOD = 3.3 aM) and 101-nucleotide NSCLC ctDNA from 200 aM to 20 nM (LOD = 5 fM) within 20 min, with clear mismatch discrimination. Notably, the interconnected Au@MNP network formed under combined magnetic and electric fields enhances redox efficiency and signal output by up to 60-fold, achieving 94% concordance with next-generation sequencing across 25 NSCLC samples, highlighting its robustness for rapid, minimally invasive cancer diagnostics (Fig. 3b) [28].

In contrast to magnetic isolation, surface-engineering–based antifouling strategies achieve matrix compatibility by chemically suppressing nonspecific adsorption at the sensor interface. A representative surface plasmon resonance imaging (SPRI) biosensing platform was developed for direct detection of KRAS p.G12D and p.G13D ctDNA mutations in plasma, which are prevalent in colorectal cancer (CRC) and associated with resistance to EGFR inhibitors. The sensing surface employs a poly-L-lysine (PLL) layer as a cationic scaffold to immobilize capture probes with high stability while enabling electrostatic tuning. To counteract nonspecific plasma protein adsorption, the PLL layer is further modified with a CEEEEE oligopeptide, whose anionic glutamate residues neutralize excess surface charge, forming a mixed-charge antifouling interface that suppresses plasma protein binding by approximately 90% in 10% diluted plasma. Peptide nucleic acids (PNAs), including PNA-WT, PNA-G12D, and PNA-G13D, serve as capture probes; their electrically neutral backbone enables highly precise single-nucleotide discrimination. Signal amplification is achieved using AuNP@KRAS conjugates (0.1 nM), which enhance refractive index shifts (Δ%R), resulting in an ultralow detection limit of 2.5 aM (5 pg µL⁻¹). The platform was validated in CRC plasma samples with a 44% variant allele frequency (VAF) without DNA isolation or PCR, requiring only 10 min of preprocessing and 5 µL of sample, and achieved 95% specificity and 85% sensitivity, demonstrating strong applicability for real-time monitoring in complex biological matrices (Fig. 3c) [45, 66].

A third matrix-compatibility paradigm relies on enzyme protection and confinement within nanoreactors, exemplified by label-free electrochemical biosensors based on glucose oxidase (GOx), encapsulated ZIF-8 metal-organic framework (MOF) nanoparticles. ZIF-8, composed of zinc ions coordinated with 2-methylimidazole ligands, forms a highly porous structure that supports high enzyme loading, preserves enzymatic activity in serum, and facilitates diffusion of small molecules such as glucose and O₂. AuNPs are electrostatically adsorbed onto the MOF surface to enhance electron transfer and anchor three-dimensional DNA walker architectures, dynamic DNA nanomachines that repeatedly traverse target molecules to amplify signals. In the presence of target miRNA-21, the DNA walker is activated and immobilizes G-quadruplex/hemin DNAzymes on the electrode surface. These DNAzymes catalyze the oxidation of ABTS into ABTS⁺, generating an electrochemical signal measured by differential pulse voltammetry (DPV), with a reproducibility of 5.1% RSD. Owing to the protective and diffusion-friendly environment of ZIF-8, this platform achieves a detection limit of 29 pM over a dynamic range of 0.1 nM to 10 µM in serum samples (Fig. 3d) [67].

A closer comparison of these matrix-compatible designs shows that they mitigate biological interference through fundamentally different mechanisms, which directly influences how broadly each approach can be applied. Magnetic nanoparticle–based systems, such as Au@MNPs, improve performance by physically extracting and concentrating ctDNA from the surrounding biofluid; as a result, their effectiveness is largely independent of surface chemistry and remains robust even in highly complex samples such as whole blood. In contrast, antifouling surface-engineering strategies rely on carefully balanced interfacial charge and probe organization to suppress nonspecific adsorption. While this enables exceptional sensitivity and single-base discrimination, the antifouling performance is intrinsically linked to surface composition and buffer conditions, which may require re-optimization when transferred to different sensing platforms. Nanoreactor-based designs, exemplified by ZIF-8 MOFs, address matrix effects by protecting enzymatic and catalytic components from deactivation while allowing controlled mass transport; however, this advantage is specific to amplification schemes that depend on enzyme activity and is less readily extended to non-enzymatic detection formats. Taken together, these approaches illustrate that matrix compatibility arises from distinct, mechanism-dependent design choices, and that optimal platform selection depends on the dominant source of interference in the target biofluid [28, 45, 67].

#### Combining Key Strategies to Improve ctDNA Detection

In this study, three key components, signal amplification, target capture, and matrix compatibility, were integrated to improve the detection of ctDNA and related biomarkers. Each method has individually proven effective: signal amplification enhances sensitivity, target capture boosts specificity, and matrix compatibility addresses the complexities of biological samples, ensuring more reliable results. Combining these approaches significantly improves overall assay efficiency and accuracy.

In a lung cancer study, Fe₃O₄@Au immunomagnetic probes combined with Au@Ag core–shell nanoparticles (SERS nanotags) enabled amplification-free, bisulfite-free detection of DNA methylation at the SHOX2 and RASSF1A genes using a multiplex SERS immunoassay. Target capture selectively enriched methylated DNA markers (m-SHOX2 and m-RASSF1A) from fragmented genomic DNA in FFPE tissues (formalin-fixed, paraffin-embedded) using Fe₃O₄@Au probes functionalized with 5-methylcytosine (5mC) antibodies. These antibodies specifically recognize methylated cytosine residues, enabling selective binding and isolation of methylated DNA fragments and improving assay specificity by discriminating them from unmethylated DNA. Signal amplification was achieved using Au@Ag SERS nanotags carrying Raman reporter molecules. MBA (4-Mercaptobenzoic Acid) served as a reporter for m-SHOX2 (~ 1079 cm⁻¹), while TFMBA (a trifluoromethyl-substituted benzoic acid derivative) served as a reporter for m-RASSF1A (~ 1630 cm⁻¹). These reporters produce strong, unique Raman signals upon laser excitation, enabling highly sensitive, simultaneous detection of multiple methylated DNA targets. This strategy achieved limits of detection of 0.52 pM for m-SHOX2 and 0.66 pM for m-RASSF1A, showing strong correlation with qPCR across 35 clinical samples. Performance metrics included 96% sensitivity, 90% specificity, and an AUC of 0.972 based on random forest analysis, a robust machine learning method for classifying complex datasets [68].

Overall, this platform provides a portable, efficient, and highly accurate approach for early-stage epigenetic profiling, and Table 1 presents a broader overview of this triple nanoparticle-based diagnostic approach. Supplementary Table S1 presents an expanded overview of nanoparticle-based ctDNA platforms, combining previously mentioned and additional entries for a more complete picture.

**Table 1:** Comparison of nanoparticle-based ctDNA platforms in terms of signal amplification, target capture efficiency, and sample compatibility

Platform / Strategy	Dominant Strategy*	Signal Modality	Key Nanomaterial(s)	LOD	Linear Range	Assay Time	Matrix	Clinical Challenge Addressed	Biomarker / Mutation	Ref
COF–AuNP / MOF–AuNP sandwich	Signal amplification	Electrochemical (Prussian blue)	COF–AuNP, Fe-MOF	7.65 fM	100 fM–100 nM	—	Serum	Low ctDNA abundance	*EGFR* L858R (NSCLC)	[39]
COF–AuNP + Au@Pt DPV	Signal amplification	Electrochemical (DPV)	Au@Pt nanohybrids	0.6 pM	—	—	Serum	Single-base discrimination	*EGFR* T790M	[51]
DNA nanosphere–Ag⁺/CuS	Signal amplification	Electrochemical (cation exchange)	DNA nanospheres, CuS	0.3 aM	1 aM–1 fM	—	Serum	Ultra-low abundance ctDNA	*EGFR* L858R	[46]
HAC–AuPt	Signal amplification	Electrochemical (H₂O₂ reduction)	AuPt alloy NPs	36 aM	10⁻¹⁶–10⁻⁸ M	—	Serum	Early-stage detection	Breast cancer ctDNA	[49]
Ag@N, O–C broom-like	Signal amplification	Optical (SERS)	AgNPs	10 aM	—	—	Plasma	Complex matrix	*PIK3CA* E545K	[52]
CdS QDs	Signal amplification	Optical (ECL)	CdS QDs	3.5 aM	—	—	Serum	Short ctDNA fragments	*EGFR* T790M	[54]
CuNP–hmC TACN	Signal amplification	Optical (fluorescence)	CuNPs	64 pM	—	—	Serum	Epigenetic modification	5hmC	[55]
UCNPs	Signal amplification	Optical (UCL)	UCNPs	6.30 pM	—	—	Serum	Autofluorescence suppression	*PIK3CA*	[56]
mAC–DC	Signal + capture	Optical (plasmonic digital)	Fe₃O₄@Au MPNPs	61.9 aM	100 aM–10 pM	1 min	Serum	Diffusion-limited capture	miR-375	[57]
Fe₃O₄@SiO₂ microfluidic	Target capture	—	Fe₃O₄@SiO₂	—	—	—	Plasma	Size-selective enrichment	ctDNA (~ 2.6 nm)	[27]
Fe₃O₄–citric acid + DPV	Target capture	Electrochemical	Fe₃O₄–CA	9 × 10⁻⁵ ng/mL	—	—	Plasma	Methylation-specific capture	*CDH1* promoter	[63]
Fe₃O₄@SiO₂ core–shell	Target capture	—	Fe₃O₄@SiO₂	—	—	—	Plasma	Short fragment isolation	*BRCA2*, IDH1	[64]
Dyna@MIX + SPRI	Capture + signal	Optical (SPRI)	Superparamagnetic beads	0.5 aM	—	< 2 h	Plasma	PCR-free SNV detection	*KRAS* G13D	[65]
Au@MNP–MB	Matrix compatibility	Electrochemical	Au@MNPs	3.3 aM	2 aM–20 nM	20 min	Whole blood	Interference resistance	NSCLC ctDNA	[28]
PLL–AuNP SPRI	Matrix compatibility	Optical (SPRI)	PLL-modified AuNPs	2.5 aM	—	15 min	Plasma	Antifouling interface	*KRAS,* G12D/G13D	[45, 66]
ZIF-8–GOx–DNA walker	Matrix compatibility	Electrochemical (DPV)	MOF–AuNP–GOx	29 pM	0.1 nM–10 µM	—	Serum	Enzyme protection	miRNA-21	[67]
Fe₃O₄@Au + Au@Ag SERS	Integrated	Optical (SERS)	Fe₃O₄@Au, Au@Ag	0.52–0.66 pM	—	—	FFPE tissue	Multiplex epigenetics	*SHOX2*,* RASSF1A*	[68]

### Fetal abnormalities and prenatal diagnostics

Nanoparticle-enabled analysis of fetal cfDNA presents a diagnostic paradigm that is fundamentally distinct from oncology-focused ctDNA detection. Cell-free fetal DNA (cffDNA), detectable in maternal circulation from as early as 4 weeks of gestation and accounting for approximately 5–20% of total cfDNA in maternal plasma, serves as a critical biomarker for non-invasive prenatal screening of fetal sex, monogenic disorders, and pregnancy-related complications [69, 70]. Despite its biological specificity, the relatively low abundance and fragmented nature of cffDNA pose significant challenges for clinical sensitivity and specificity, particularly due to dilution by maternal cfDNA, necessitating advanced analytical strategies to ensure reliable fetal genetic profiling [5]. In this context, diagnostic accuracy in prenatal testing relies primarily on absolute specificity rather than extreme sensitivity, as even minimal false-positive signals may lead to incorrect sex determination or misclassification of chromosomal abnormalities. Accordingly, nanoparticle-based platforms for fetal diagnostics emphasize stringent probe complementarity, controlled probe density, and minimized background signal, either by enhancing cffDNA isolation or amplifying detection signals from complex biofluids such as maternal plasma, blood, or urine. These approaches reduce reliance on invasive procedures such as amniocentesis, which carry an inherent risk of miscarriage [71]; however, broader clinical translation and scalability across diverse populations require further validation to minimize potential ethnic bias in genetic screening (Table 2).

#### Fetal sex determination

**Fig. 4:**
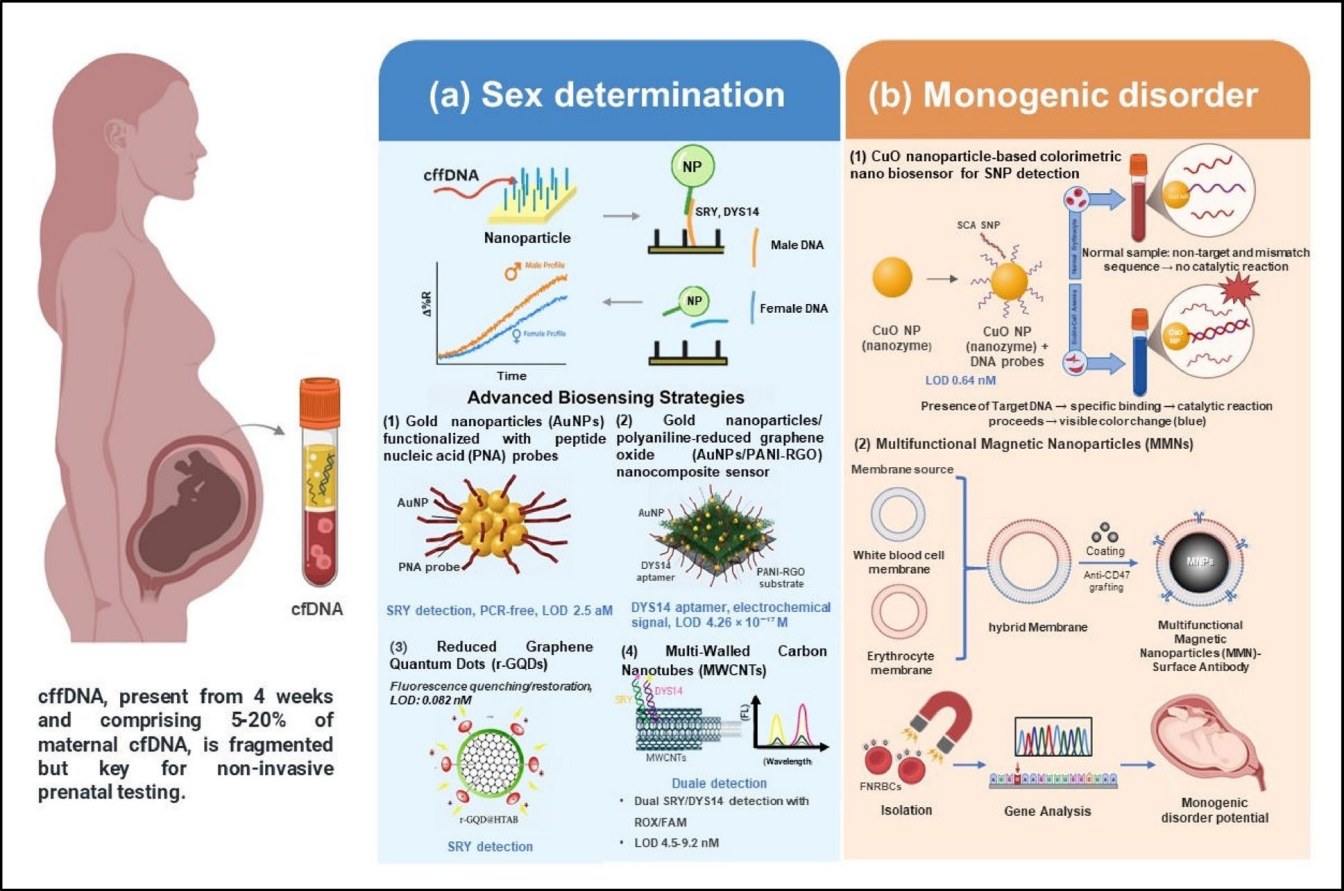
Nanoparticle-enabled enhancement of cell-free fetal DNA detection. Nanoparticle-assisted strategies to improve the detection and analysis of cell-free fetal DNA (cffDNA), which constitutes only 5–20% of maternal cfDNA and is highly fragmented. (**a**) For fetal sex determination, gold nanoparticles (AuNPs) functionalized with peptide nucleic acid (PNA) probes leverage surface plasmon resonance to detect the SRY gene. AuNPs embedded within polyaniline–reduced graphene oxide (PANI–RGO) composites amplify electrochemical signals for DYS14 detection. Reduced graphene quantum dots (r-GQDs) enable fluorescence quenching/restoration for SRY sensing, while multi-walled carbon nanotubes (MWCNTs) utilize π–π stacking for multiplex detection of SRY and DYS14. (**b**) For monogenic disease diagnosis, copper oxide nanoparticles (CuO NPs) provide peroxidase-like catalytic activity for rapid colorimetric detection of sickle cell anemia SNPs. Multifunctional magnetic nanoparticles (MMNs) coated with leukocyte/erythrocyte hybrid membranes selectively capture fetal nucleated red blood cells (FNRBCs), enabling downstream genotyping via digital PCR or next-generation sequencing. Created in BioRender. Schwaminger, S. (2026) https://BioRender.com/s25hisi

In fetal sex determination, specific Y-chromosome sequences are targeted using nanoparticle platforms that bypass PCR amplification. The *SRY* (sex-determining region Y) gene, a key regulator located on the Y chromosome that initiates male sex development by triggering testicular differentiation, and the DYS14 marker, a repetitive DNA sequence unique to the Y chromosome used as a reliable indicator of male fetal DNA, are detected to accurately determine fetal sex. This approach minimizes artifacts and reduces diagnostic times while achieving aM sensitivity (Fig. 4a; Table 2).

For example, AuNPs functionalized with short peptide nucleic acid (PNA) probes complementary to the *SRY* gene enable hybridization with target DNA in maternal plasma. Changes in the nanoparticles’ optical properties, particularly surface plasmon resonance, are detected by imaging systems, allowing accurate fetal sex identification at concentrations as low as 2.5 aM with complete diagnostic accuracy [72].

In a related approach, AuNPs were integrated into electrochemical sensors constructed on polyaniline and reduced graphene oxide substrates. The biosensor, named Apt/PANI, RGO, GNPs/Au, incorporated a *DYS14* aptamer, a single-stranded DNA sequence that specifically binds to the Y-chromosome’s *DYS14* target, enabling early fetal sex determination in maternal blood. Polyaniline (PANI) and reduced graphene oxide (RGO) enhanced the sensor’s conductivity and surface area, while gold nanoparticles amplified the electrochemical signal. This label-free sensor detected cffDNA by measuring disruptions in electrical current upon *DYS14* hybridization, achieving a detection limit of 4.26 × 10⁻¹⁷ M within a linear range of 1.0 × 10⁻¹⁶ to 1.0 × 10⁻⁸ M. With high selectivity against mismatched DNA and a relative standard deviation of 2.70% after four sensor reuse cycles, this biosensor provided a sensitive, cost-effective, and non-invasive alternative to traditional prenatal testing methods like amniocentesis [73]. These methods work by converting the recognition of DNA into a measurable physical signal, including optical or electrical, thereby eliminating the need for complex amplification steps such as PCR.

Fluorescence-based methods have also been employed, where reduced graphene quantum dots (r-GQDs) were used as fluorescence quenchers. In the absence of target DNA, the fluorescence of a reporter probe remained suppressed; when hybridization with *SRY* cffDNA occurred, the signal was restored and measured with high sensitivity in plasma samples [74].

Multi-walled carbon nanotubes (MWCNTs) exploit π–π stacking interactions, where the aromatic rings of DNA probes non-covalently align with the conjugated carbon surfaces of the nanotubes through planar, attractive interactions. This enhances the binding specificity and stability of the probes, enabling the simultaneous detection of *SRY* and *DYS14* sequences using fluorescent dyes ROX and FAM. ROX and FAM act as distinct reporters, with each labeling a specific target, allowing multiplexed detection in a single assay. This approach achieves fluorescence recovery of 79.5–81.5% and limits of detection (LODs) of 4.5–9.2 nM in clinical samples, demonstrating its potential for multiplexed DNA screening. However, careful calibration is required to avoid cross-reactivity when analyzing heterogeneous sample matrices [71].

#### Monogenic disorders detection

Monogenic disorders are caused by mutations in a single gene, resulting in a specific inherited condition. In the detection of such disorders, including sickle cell anemia (SCA), a blood disease caused by a single nucleotide polymorphism (SNP) in the β-globin gene (adenine [A] replaced by thymine [T], leading to a valine substitution for glutamic acid in hemoglobin), a colorimetric nanobiosensor was developed by Mahsa Kasiri and Mahdi Rahaie using copper oxide (CuO) nanoparticles. These nanoparticles act as a nanoenzyme, mimicking the activity of natural peroxidase to catalyze chemical reactions without requiring protein enzymes. The plate- or straw-shaped CuO nanoparticles (diameter 21.65–49.84 nm, surface area 28.978 m²/g, pore diameter ~ 13 nm) were combined with single-stranded DNA (ssDNA) probes complementary to the SCA-specific sequence in cell-free fetal DNA. Upon hybridization with perfectly matched target sequences to form double-stranded DNA (dsDNA), the nanoparticles exhibited enhanced peroxidase-like catalytic activity, which catalyzed the oxidation of tetramethylbenzidine (TMB) by H₂O₂, producing a visible blue color detectable at 652 nm. This enzyme-free nanoenzyme assay selectively discriminated the target SNP from non-target or mismatched sequences (one or two base differences), even in healthy female plasma. It achieved a detection limit of 0.64 nM, a linear range of 2–12 nM (R² = 0.96), and produced results within 10 min, offering a rapid, low-cost, visible, and non-invasive prenatal diagnostic tool for SCA and potentially other hereditary diseases (Fig. 4b; Table 2) [75].

Multifunctional magnetic nanoparticles (MMNs) coated with leukocyte and erythrocyte derived membranes are engineered to mimic fetal cell surface markers, including CD47, which serves as a “self” signal to evade phagocytosis and enhance selective binding to fetal nucleated red blood cells (FNRBCs) in maternal blood. This biomimetic design enables the efficient isolation of FNRBCs via magnetic separation while minimizing maternal cell contamination. The captured FNRBCs can then be analyzed using digital PCR or sequencing, achieving 100% concordance with invasive diagnostic methods and enabling accurate detection of monogenic disorders in a non-invasive manner. While this approach shows great promise for prenatal diagnostics, variability in membrane coating can affect reproducibility, highlighting the need for standardized manufacturing protocols to ensure regulatory compliance and consistent clinical performance. (Fig. 4b; Table 2) [76].

Collectively, these studies illustrate how nanoparticle design parameters are deliberately tuned to meet the distinct diagnostic constraints of fetal cfDNA analysis, in contrast to oncology-oriented ctDNA detection discussed in earlier sections. As summarized in Table 2, prenatal applications prioritize absolute sequence specificity, controlled probe density, and stringent suppression of background signals to ensure reliable discrimination of fetal genetic signatures within a predominantly maternal cfDNA pool. This design philosophy is reflected in Fig. 4, where nanoparticle properties such as surface functionalization, binding affinity, and signal-to-noise optimization are directly linked to diagnostic outcomes, including accurate fetal sex determination and chromosomal abnormality detection. In comparison, oncological platforms emphasize extreme sensitivity and mutation enrichment to resolve rare tumor-derived variants against a high background of wild-type DNA. Together, these different design approaches underscore an integrated framework in which nanoparticle-enabled cfDNA sensing is not universally optimized, but instead rationally adapted to the biological context and clinical question at hand.

**Table 2:** Nanoparticle properties for fetal abnormalities and prenatal diagnostics

Nanoparticle	Key Properties	Disease/ Condition	Detection Method	Quantitative Data	Efficacy/Accuracy	Sample	Ref.
Multi-Walled Carbon Nanotubes (MWCNTs)	Fluorescence quenching, π-stacking	Fetal Sex Determination	Fluorescence	LOD: 4.5 nM (*SRY*), 7.6 nM (*DYS14*); Linear range: 0.01 to 1 µM (*SRY*), 0.02 to 1 µM (DYS14); Recovery: 79.5 to 81.5%	42-fold (*SRY*), 20-fold (*DYS14*) perfect match enhancement	Maternal Plasma	[71]
Magnetic Membrane Nanoparticles (MMNs)	339.9 nm, superparamagnetic, hybrid membrane	Monogenic Disorders	Magnetic Separation, Sequencing, ddPCR	FNRBC isolation: >90% efficiency/purity; FNRBC count: 37.6 ± 36.0 cells/mL (2nd trimester)	100% concordance (5/5 cases)	Maternal Blood	[76]
Gold Nanoparticles (AuNPs)	10–20 nm, LSPR	Fetal Sex Determination	surface plasmon resonance imaging (SPRI)	LOD: 2.5 aM; Δ%R male/Δ%R female > 1, CV%: 27%; Assay time: 70 min	100% accuracy	Maternal Plasma	[72]
Reduced Graphene Quantum Dots (r-GQDs), Hexadecyl Trimethyl Ammonium Bromide (HTAB)	< 10 nm, fluorescence, cationic charge	Fetal Sex Determination	Fluorescence	LOD: 0.082 nM; Linear range: 0.16 to 1.5 nM; R²: 0.99	High specificity in spiked plasma	Maternal Plasma	[74]
Copper (II) Oxide Nanoparticles (CuO)	21.65–49.84 nm, peroxidase activity, 28.978 m²/g	Sickle Cell Anemia	Colorimetric	LOD: 0.64 nM; Linear range: 2 to12 nM; R²: 0.96; Time: 10 min	High specificity in plasma	Maternal Plasma	[75]
AuNPs, Reduced Graphene Oxide (RGO)	AuNPs: 10–20 nm, conductivity; RGO: large surface area	Fetal Sex Determination	Square Wave Voltammetry (SWV)	LOD: 4.26 × 10⁻¹⁷ M; Linear range: 1.0 × 10⁻¹⁶–1.0 × 10⁻⁸ M; R²: 0.991; Stability: 95.01%	Good PCR agreement, high selectivity	Maternal Plasma	[73]

## Nanoparticle-based cfDNA modulation for advanced therapeutic interventions

CfDNA, once regarded as a passive marker of cell death, is now increasingly recognized as a potent mediator of inflammation across a broad range of pathologies, including autoimmune diseases, sepsis, cancer, and localized inflammatory conditions.

A promising therapeutic strategy has emerged in recent years: neutralizing cfDNA at its source. NPs, particularly those with cationic surfaces, offer a unique platform to scavenge cfDNA via strong electrostatic or chemical interactions, thereby halting downstream immune activation [76]. Many of these NPs are multifunctional combining cfDNA scavenging with drug delivery, immune modulation, or tissue targeting to maximize therapeutic outcomes [62, 77–79].

### Rheumatoid arthritis (RA)

**Fig. 5:**
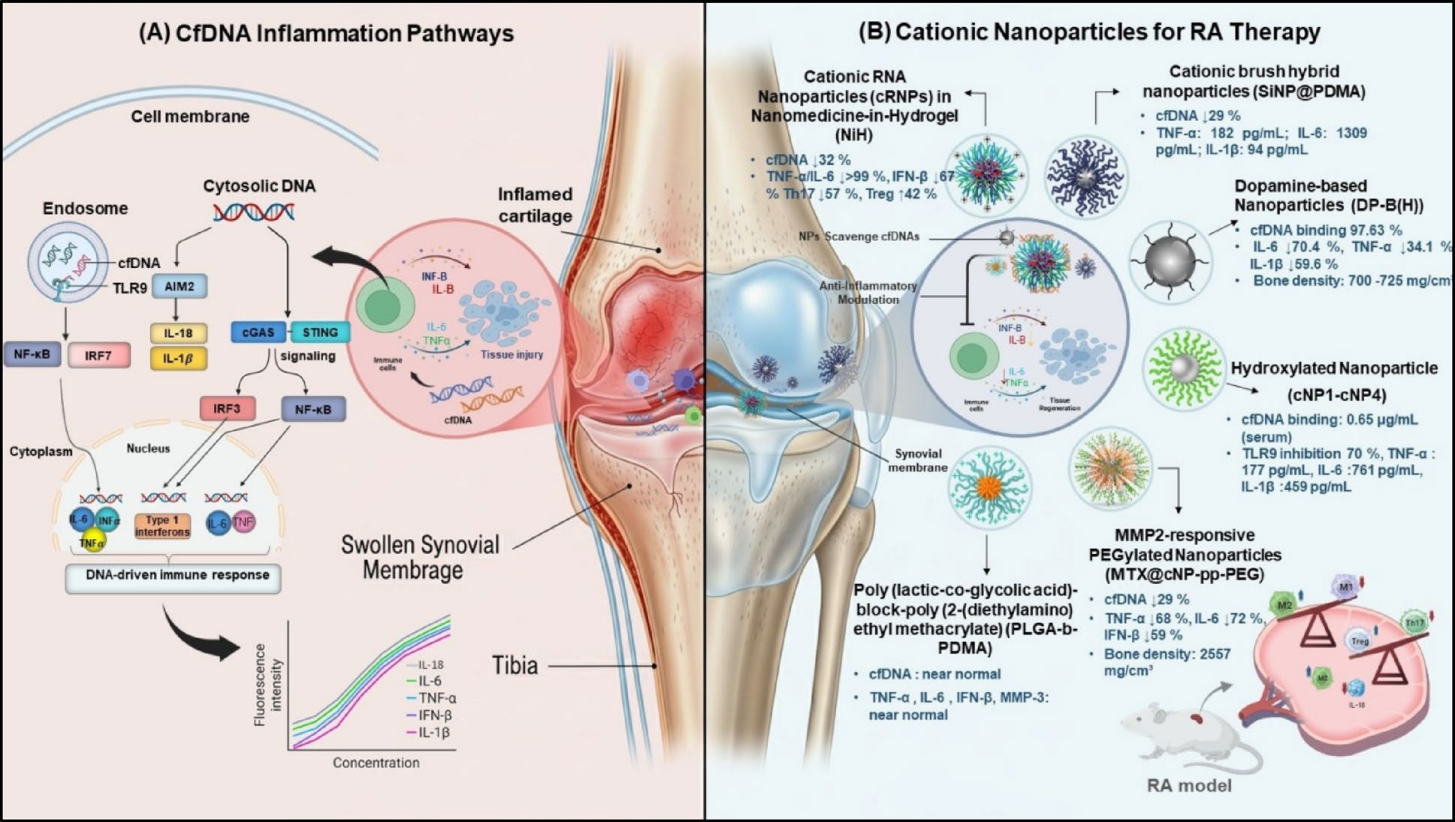
Role of cfDNA in rheumatoid arthritis and therapeutic action of cationic nanoparticles. cfDNA-mediated inflammatory signaling in rheumatoid arthritis (RA) and the therapeutic functions of cationic nanoparticles (cNPs). (**a**) cfDNA released from apoptotic or necrotic cells enters synovial cells through endocytosis, activating the TLR9 or cGAS–STING pathways and inducing pro-inflammatory cytokines (TNF-α, IL-6, IFN-β). These responses contribute to cartilage degradation, bone erosion, and immune dysregulation characterized by increased Th17 activity and reduced Treg and M2 macrophage populations. (**b**) In collagen-induced arthritis (CIA) models, cNPs (40–100 nm, + 5 to + 22.8 mV) neutralize cfDNA through electrostatic interactions. Nanomedicine-in-Hydrogel (NiH) reduces cytokine levels by > 99%; dopamine-based nanoparticles (DP-B(H)) bind 97.6% of cfDNA and lower IL-6 by 70%; and MMP2-responsive PLGA-b-PDMA nanoparticles reduce paw swelling by 44–50%, restore bone density, and improve mobility, providing disease-modifying therapeutic effects with minimal toxicity at optimized doses. Created in BioRender. Schwaminger, S. (2026) https://BioRender.com/ul19n7q

RA is a chronic autoimmune disease characterized by persistent joint inflammation, resulting in cartilage and bone destruction. A major driver of this inflammation is the accumulation of cfDNA in the synovial fluid, which acts as a danger-associated molecular pattern (DAMP) to activate innate immune responses. cfDNA can be internalized into endosomes, where it is recognized by TLR9, triggering IRF7 and NF-κB signaling and inducing type I interferon (IFN-α) production. In the cytosol, cfDNA is sensed by cyclic GMP–AMP synthase (cGAS), which synthesizes cGAMP to activate STING (stimulator of interferon genes), leading to recruitment of IRF3 and NF-κB and promoting type I interferon (IFN-β) production along with pro-inflammatory cytokines such as TNF-α and IL-6. Furthermore, cytosolic double-stranded DNA can activate the AIM2 inflammasome, resulting in caspase-1–mediated maturation of IL-1β and IL-18 and triggering inflammatory cell death (pyroptosis), which further amplifies joint inflammation (Fig. 5a) [80, 81].

To counter these responses, cationic nanoparticles (cNPs) have been developed to bind and neutralize cfDNA, thereby dampening both endosomal and cytosolic DNA-sensing pathways. In collagen-induced arthritis (CIA) models, cNPs have reduced cytokine production and improved joint pathology, demonstrating their therapeutic potential. Nonetheless, challenges remain regarding long-term safety, immune homeostasis, and clinical translation, emphasizing the need for careful evaluation of cfDNA-targeted therapies in RA [6, 82].

One innovative approach employs cationic RNA Nanoparticles (cRNPs) in Nanomedicine-in-Hydrogel (NiH), in which cNPs (~ 51 nm, + 5.2 mV), self-assembled from amphiphilic miktoarm star polymers, scavenge negatively charged cfDNA to prevent its uptake by immune cells and activation of DNA sensors such as cGAS. Simultaneously, RU.521-loaded nanoparticles (~ 63 nm, + 6.2 mV) deliver the selective small-molecule cGAS inhibitor RU.521 to immune sites. RU.521 binds the cGAS/dsDNA complex, blocking cGAMP production and downstream STING activation, suppressing type I interferon responses. Both nanoparticles are embedded in a polyethylene glycol (PEG) hydrogel, which prolongs retention in lymph nodes. In CIA mice, this system reduced cfDNA by 32%, decreased TNF-α and IL-6 by over 99% and IFN β by 67%, lowered Th17 cells by 57%, increased protective regulatory T cells (Tregs) by 42%, and promoted tissue-healing M2 macrophages, effectively breaking the inflammatory cycle and outperforming standard therapies [83].

Cationic brush hybrid nanoparticles, termed silica nanoparticles grafted with poly(2-(dimethylamino) ethyl methacrylate (SiNP@PDMA), with tunable particle diameters of 50, 100, and 200 nm and degrees of polymerization (DP) of 100, 200, and 300, were developed as cfDNA scavengers to suppress aberrant immune activation and alleviate inflammation in rheumatoid arthritis. These core–shell nanoparticles were fabricated via surface-initiated atom transfer radical polymerization (ATRP) on silica cores, enabling precise control over particle size and cationic polymer density. Among the series, SiNP@PDMA with a 100 nm diameter and DP = 200 exhibited superior accumulation and prolonged retention within the inflamed joint cavity, along with the highest binding affinity for cfDNA. This resulted in effective inhibition of inflammatory cell infiltration, pannus formation, and cartilage and bone destruction, leading to a significant reduction in hind paw swelling in arthritis models. Increasing the cationic content of SiNP@PDMA (from DP = 100 to DP = 300) enhanced electrostatic interactions with anionic cfDNA, further augmenting anti-inflammatory efficacy. The 100 nm SiNP@PDMA achieved an optimal balance between blood circulation time, localized retention in inflamed sites, and biosafety. Compared with conventional cationic micelles, these hybrid nanoparticles offer greater structural stability, reduced cytotoxicity, and tunable physicochemical properties, providing a promising platform for safe and effective cfDNA-scavenging therapy in rheumatoid arthritis [84].

Another promising development is the use of positively charged polydopamine nanoparticles (DP-B(H), + 10.33 mV), which are biodegradable and safe. In CIA rats, these particles bound 97.63% of cfDNA, reduced joint swelling to a score of 2.3, and lowered IL-6 by 70.4%, 34.1% in TNF-α, and 59.6% in IL-1β. Over 27 days, they improved bone density from 700 mg/cm³ to 725 mg/cm³ (about 3–4%) with no major side effects, though their long-term impact on joints requires further study [85].

Xingliang Liu and colleagues demonstrated that nanoparticles entering the bloodstream are rapidly coated by serum proteins, forming a protein corona that can dramatically alter their biodistribution, cellular uptake, and therapeutic activity. To address this, they engineered a series of cNPs specifically designed to capture harmful cfDNA, a key driver of RA progression. The most effective formulation, cNP2, featured secondary amines and a dual hydroxyl shell, which provided strong cfDNA binding (BE50 = 0.17 µg/mL in buffer; 0.65 µg/mL in serum) and efficiently inhibited TLR9 signaling by 70% at low doses. In RA rat models, cNP2 exhibited double the accumulation in inflamed joints, reduced joint cfDNA levels from 6.1 to 2.2 µg/mL, and normalized key inflammatory markers (TNF-α: 177 pg/mL, IL-6: 761 pg/mL, IL-1β: 459 pg/mL). Moreover, it fully reversed paw swelling from 3.3 mL back to baseline. Mechanistic studies showed that the protein corona of cNP2 contained 1.4–1.5 times fewer immune-tagging proteins (e.g., IgM and complement), which enhanced circulation time and joint targeting, highlighting how smart surface engineering of nanoparticles can optimize both safety and therapeutic efficacy in RA [86].

A smart nanoparticle system that combines cfDNA scavenging with methotrexate (MTX) delivery, termed MMP2-responsive PEGylated cationic nanoparticles (MTX@cNP-pp-PEG), has also been developed. These nanoparticles (67–74 nm) feature a PLGA core encapsulating 93.7% of MTX and a cationic PDMA shell, with a zeta potential that increases from + 13.7 mV to + 22.8 mV upon MMP2 activation, and a hydrodynamic size of 145 nm. In collagen-induced arthritis rats, administered every two days at 1 mg/kg MTX and 25 mg/kg cNP, this system reduced paw swelling by 44%, lowered joint cfDNA from 9.79 to 4.16 µg/mL, restored bone density to 2557 mg/cm³, and preserved 82% of cartilage without detectable organ toxicity. It also significantly suppressed pro-inflammatory cytokines: TNF-α by 68%, IL-6 by 72%, and IFN-β by 59%. Importantly, this system differs from the previously described cNP2, which focuses solely on cfDNA capture without drug loading. Both nanoparticle strategies should be compared in figures to highlight their complementary mechanisms. Long-term joint outcomes and safety of MTX@cNP-pp-PEG require further investigation [87].

Another system using PLGA-b-PDMA nanoparticles (~ 40 nm) reduced inflammation by blocking cfDNA from activating TLR9 in joint cells. In collagen-induced arthritis models, doses of 12.5 to 25 mg/kg reduced ankle swelling by 50% (from 2.4 mm to 1.7 mm in mice) and increased bone density to 1442 mg/cm³ in rats, restoring partial mobility. However, high doses (25 mg/kg) caused a 25% mortality rate in rats, and the lack of human data or comparisons with treatments like methotrexate raises concerns about clinical use [29].

The result is a clear suppression of cfDNA-triggered inflammation, with outcomes including clinical score improvements, reduced cytokine expression, and protection of joint structure (Fig. 5b). Table 3 and Supplementary Table S2 provide a more detailed summary and comparison of these cNPs in managing RA.

**Table 3:** Cationic nanoparticles for cfDNA scavenging in rheumatoid arthritis therapy

Nanoparticle Type	Composition	Size (nm)	ZP (nm)	cfDNA Reduction	Inflammation Reduction	Clinical Score	Additional Outcomes	Key Feature	Ref.
Cationic Nanoparticles (cNPs)/cationic RNA Nanoparticles (cRNPs) in Nanomedicine-in-Hydrogel (NiH)	Poly (ethylene glycol)-poly (dimethylaminoethyl methacrylate)-poly(2-(diisopropylamino) ethyl methacrylate) (PEG-PDMA-PDPA) + RU.521 (a small-molecule inhibitor of cGAS)	51 (cNPs), 63 (cRNPs)	+ 5.2 (cNPs), + 6.2 (cRNPs)	32% (serum)	Tumor Necrosis Factor-alpha (TNF-α)/Interleukin-6 (IL-6) > 99%, Interferon-beta (IFN-β) 67%	Reduced severity	Regulatory T cells (Treg) + 42%, M2/M1 increase, Lymph Node (LN) retention 7.8-fold	Hydrogel+CGAS inhibitor	[83]
Dopamine-based Nanoparticles (DPs) (DP-B(H))	Polydopamine with dimethylamino groups	Nano range	+ 10.33	High binding 97.63%	IL-6 70.4%	2.3	Bone density 725 mg/cm³	Biodegradable polydopamine	[85]
Hydroxylated cationic nanoparticles (cNP1, cNP2, cNP3, cNP4)	Poly(ε-caprolactone) (PCL)-based di-block copolymers with hydroxyl and amine groups (e.g., PCL60-b-PGDMEN150, PCL60-b-PGEA150, PCL60-b-PGEDA150, PCL60-b-PDMA150)	40 ± 5	+ 15 to + 18	High (cNP2), Moderate (cNP1, cNP3), Low (cNP4)	High (cNP2), Moderate (cNP1, cNP3), Low (cNP4)	Not specified	Hydroxyl shell improves biocompatibility, cNP2 shows best cfDNA binding and reduced opsonin adsorption.	Dual hydroxyl shell	[86]
Cationic nanoparticle (cNP)	Poly (lactic-co-glycolic acid)-block-poly (2-(diethylamino) ethyl methacrylate) (PLGA-b-PDMA)	~ 40	+ 18	near-normal	TNF-α, IL-6, IFN-α, MMP-3 (near normal)	~ 50% swelling reduction at 12.5 mg/kg; >50% at 25 mg/kg; PDMA ~ 25%	Improved BMD (1346–1442 mg/cm³), reduced damage, better mobility (3.5 rpm), targeted joints, no toxicity	PDMA shell, local delivery	[29]
Matrix Metalloproteinase 2 (MMP2)-responsive PEGylated cationic nanoparticles (MTX@cNP-pp-PEG)	Poly (lactic-co-glycolic acid)-poly(2-(diethylamino) ethyl methacrylate)-poly (ethylene glycol) with MMP2-sensitive peptide (PVGLIG) (PLGA-b-PDMA-pp-PEG); Methotrexate (MTX) loaded in hydrophobic core	74 to 67	+ 13.7 to + 22.8	Reduced to 4.16 µg/mL (from 9.79 µg/mL in PBS control), close to normal	TNF-α: 136 pg/mL; IL-6: 870 pg/mL; IL-1β: 652 pg/mL (all close to normal)	Hind paw swelling reduced by 44% at day 28	Restored BMD (~ 2557 mg/cm³), ~ 82% cartilage retention	MMP2-responsive PEG	[87]
Cationic brush hybrid nanoparticles (SiNP@PDMA)	Silica nanoparticles grafted with poly (2-(dimethylamino) ethyl methacrylate) (SiNP@PDMA)	50, 100, 200 (core); 100 nm optimal	+ 12.4 to + 39.0 (varies with PDMA content)	To 2.4 µg/mL (SiNP100-PDMA200, from 7.1 µg/mL)	TNF-α: 182 pg/mL; IL-6: 1309 pg/mL; IL-1β: 94 pg/mL	lowest clinical scores	High joint retention, low toxicity	Tunable ploymer brush	[84]

### Sepsis

**Fig. 6:**
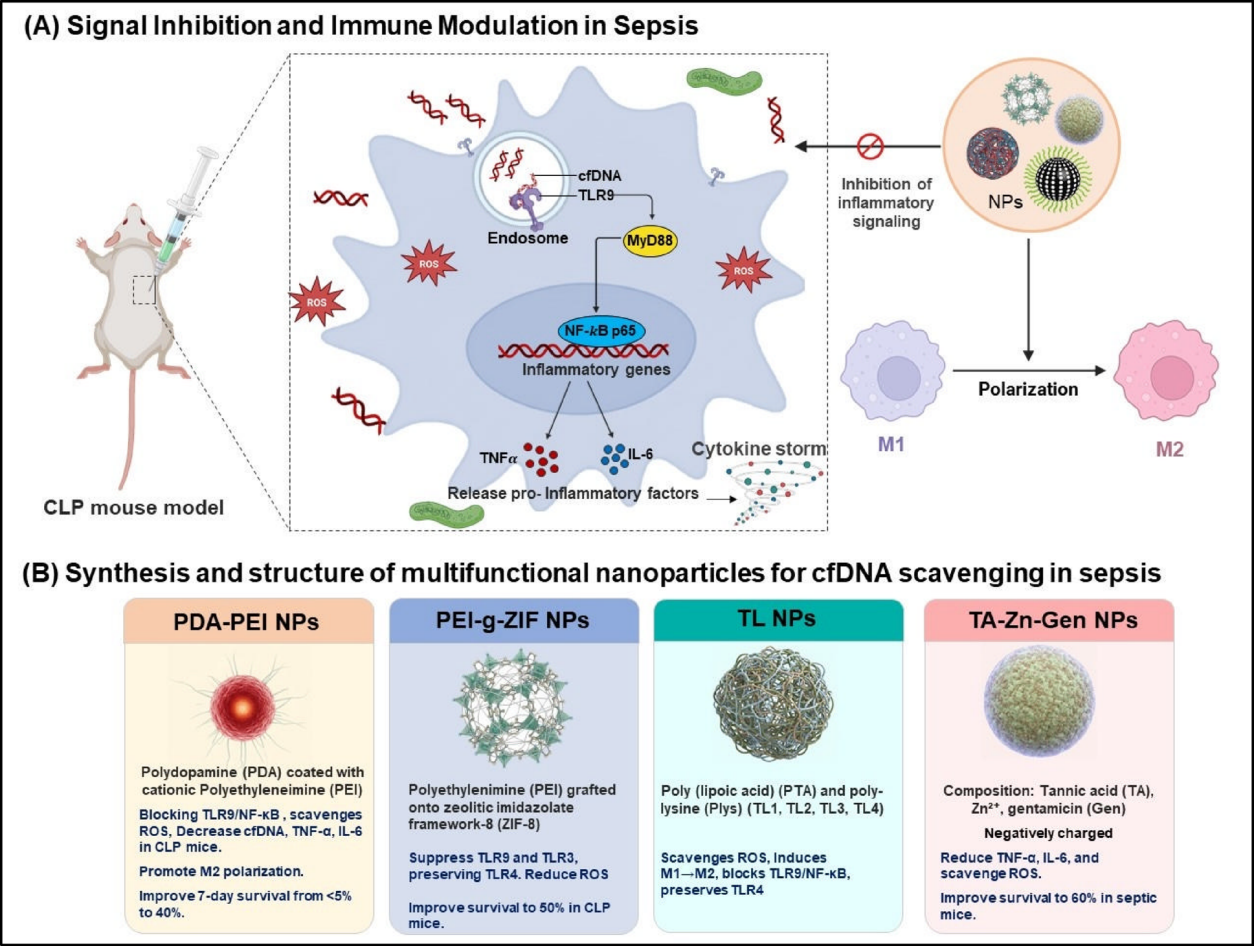
Nanoparticle-mediated cfDNA scavenging strategies for sepsis treatment. (**a**) In the cecal ligation and puncture (CLP) mouse model, cfDNA from injured host tissues or pathogens enters endosomes and activates TLR9–MyD88 signaling, inducing NF-κB p65–mediated transcription of pro-inflammatory cytokines (TNF-α, IL-6) and amplifying the cytokine storm. Nanoparticle intervention suppresses this inflammatory cascade and promotes macrophage polarization from the pro-inflammatory M1 to the anti-inflammatory M2 phenotype. (**b**) Four engineered nanoparticle systems, PDA–PEI, PEI-g-ZIF, Toll-like nanoparticles (TL-NPs), and TA–Zn–Gen nanoparticles, are shown with their compositions, mechanisms of cfDNA binding, and therapeutic activities, including cytokine reduction, immune modulation, and improved survival in sepsis models. Created in BioRender. Schwaminger, S. (2026) https://BioRender.com/qr6ueuu

In sepsis, cfDNA derived from injured host tissues or invading pathogens acts as a potent immunostimulatory molecule. Following internalization via endocytosis, cfDNA accumulates within acidified endosomes, where it is recognized by Toll-like receptor 9 (TLR9), a DNA-sensing pattern recognition receptor specialized for detecting unmethylated CpG motifs. Ligand engagement induces conformational changes that bring together the intracellular TIR domains of TLR9, enabling recruitment of myeloid differentiation primary response protein 88 (MyD88) and formation of the Myddosome signaling complex (MyD88–IRAK4–IRAK1). This cascade activates TRAF6 and culminates in the nuclear translocation of NF-κB p65, a key transcription factor that drives robust production of pro-inflammatory cytokines such as TNF-α and IL-6. The excessive cytokine output further amplifies immune signaling through STAT3 and other secondary pathways, creating a self-reinforcing inflammatory loop. Over time, this dysregulated response contributes to endothelial injury, metabolic dysfunction, and multi-organ failure, the hallmarks of severe sepsis. Therapeutically, interventions that neutralize cfDNA, inhibit endosomal TLR9 signaling, or shift macrophage polarization from M1 back toward the anti-inflammatory M2 phenotype represent promising strategies to restore immune balance and mitigate sepsis-induced organ damage (Fig. 6a) [88–92].

Polydopamine–polyethylenimine (PDA–PEI) nanoparticles alleviate sepsis through a dual anti-inflammatory mechanism. First, the polyethylenimine (PEI) component scavenges circulating cfDNA, thereby preventing its recognition by TLR9 and suppressing downstream NF-κB–mediated inflammatory signaling. Concurrently, the redox-active polydopamine (PDA) shell neutralizes excessive reactive oxygen species (ROS), reducing oxidative stress and tissue injury [89]. In a murine model of sepsis induced by cecal ligation and puncture (CLP), which mimics polymicrobial human infection, PDA–PEI treatment markedly decreased circulating cfDNA, TNF-α, and IL-6 levels, improving the 7-day survival rate from less than 5% to 40%. Furthermore, PDA–PEI nanoparticles promoted the polarization of macrophages toward the anti-inflammatory M2 phenotype, contributing to the overall suppression of hyperinflammation. In vitro, activation of the TLR9/NF-κB axis was found to elevate IL-6 secretion, which in turn drives STAT3 signaling, a pathway significantly attenuated upon IL-6 neutralization [88, 89].

Similarly, PEI-grafted zeolitic imidazolate framework-8 (PEI-g-ZIF) nanoparticles act as an effective “nanotrap” to sequester circulating cfDNA during sepsis. ZIF-8, a metal–organic framework composed of Zn²⁺ ions coordinated with 2-methylimidazolate linkers, provides a highly porous and stable crystalline structure with a large surface area for molecular adsorption. Grafting cationic polyethylenimine (PEI) onto the ZIF-8 core endows the hybrid nanoparticles with strong electrostatic affinity for negatively charged cfDNA while retaining the excellent stability and dispersibility of the MOF scaffold. These PEI-g-ZIF nanoparticles efficiently neutralize cfDNA and selectively suppress TLR9- and TLR3-dependent inflammatory signaling, yet preserve TLR4 activity, which is essential for antimicrobial defense. In a cecal ligation and puncture mouse model of sepsis, administration of PEI-g-ZIF nanoparticles improved survival to 50%, reduced oxidative stress by lowering ROS production, and restored both circulating cfDNA and pro-inflammatory cytokines to near-physiological levels, demonstrating their therapeutic potential for controlling sepsis-induced hyperinflammation [93].

Tannic acid–poly-L-lysine nanoparticles (TLs) are electrostatically assembled nanostructures generated through the interaction of negatively charged poly(lipoic acid) (PTA) and positively charged poly-L-lysine (Plys), forming stable polyelectrolyte complexes with tunable surface charge and structural integrity.Five formulations (TL1–TL4) were produced with different PTA: Plys ratios, and TL2 (~ 120 nm in size and + 25 Mv), showed the best balance of stability, cfDNA adsorption, and safety. TL2 bound > 94% of cfDNA at 4 µg/mL and maintained ~ 80% binding in 10% fetal bovine serum (FBS). It also scavenged ROS and NO, reduced bacterial burden, and shifted macrophages from a pro-inflammatory M1 state to an anti-inflammatory M2 state. TL2 suppressed TNF-α and IL-6 to near-control levels and blocked cfDNA-induced TLR9/NF-κB signaling while preserving TLR4-mediated antibacterial responses. In a CLP sepsis mouse model, TL2 markedly improved survival, normalized cfDNA and cytokines, and lowered tissue ROS. Overall, TL2 represents a biodegradable, antibiotic-free nanoplatform that simultaneously targets oxidative stress, infection, and hyperinflammation in sepsis [94].

Interestingly, tannic acid-Zn²⁺-gentamicin (TA-Zn-Gen) nanoparticles are negatively charged spherical particles formed through a one-pot process that combines tannic acid, Zn²⁺, and gentamicin. Unlike typical cationic nanoparticles that use electrostatic interactions to bind cfDNA, these nanoparticles utilize hydrogen bonding to attach cfDNA, offering a novel approach to cfDNA scavenging. This unique binding mechanism highlights that cfDNA capture is not restricted to cationic particles. The nanoparticles demonstrated superior bacterial clearance compared to free gentamicin, with significant reductions in proinflammatory cytokines such as TNF-α and IL-6, which are elevated in sepsis and contribute to the hyperinflammatory state. Additionally, they also scavenged ROS, reducing oxidative stress and preventing further cellular damage. These therapeutic effects were associated with an improved survival rate of 60% in septic mice, suggesting a promising alternative approach to managing sepsis beyond conventional treatments. This system demonstrates that alternative bonding mechanisms, such as hydrogen bonding, can be exploited to effectively target cfDNA and modulate immune responses, opening new avenues for therapeutic development in inflammatory diseases like sepsis [95]. Figure 6b; Table 4 further illustrate these findings, emphasizing the potential of non-cationic nanoparticles in sepsis treatment.

**Table 4:** Multifunctional nanoparticles for cfDNA scavenging and immune modulation in sepsis treatment

NP System	Composition		Size (nm)	ZP (mV)		cfDNA Binding	Functions	Survival (CLP)	Key Outcomes	Key Design Feature	Ref.
Polydopamine (PDA)-Polyethylenimine (PEI)		PDA + PEI	161 ± 6		+ 43.8	Outstanding	cfDNA/TLR9 inhibition, ROS scavenging, M2 shift	40% (7 days)	Reduced cytokines/organ damage (*p* < 0.05), TLR9-MyD88 suppression	Biodegradable cationic core	[89]
Polyethylenimine-grafted Zeolitic Imidazolate Framework (PEI-g-ZIF)		ZIF-8 + PEI 1800	Not specified		Not specified	High (> 1 NPs: DNA)	cfDNA/TLR9/3 inhibition, ROS reduction	50% (1 h and 12 h post-CLP)	Normalized cfDNA/TNF-α, reduced M1/ROS (*p* < 0.001)	MOF core + PEI graft	[93]
Tannic Acid–Polylysine nanoparticles (TL or TL2 system)		Poly (lipoic acid) (PTA) and poly-lysine (Plys)	100 (TL1, smallest) to ~ 200 (TL3, largest)		~+30 (TL3, highest), decreasing for TL2, TL1, lowest for TL4, TL5	> 94% (4 µg/mL) for TL2	cfDNA/LPS binding, ROS/NO scavenging, antibacterial	Significant	M2 (3.58% to 27.9%), reduced bacteria/cytokines/ROS (*p* < 0.05)	Polyphenol-polycation hybrid	[94]
Tannic Acid–Zinc–Gentamicin complex (TA-Zn-Gen)		TA + Zn²⁺ + Gen	~ 200		Negative	High (calf thymus)	cfDNA/TLR9 inhibition, ROS/NO scavenging, antibacterial	60% (5 days)	Lowest cfDNA/bacteria, organ protection, no toxicity (*p* < 0.05)	Metal-polyphenol-drug complex	[95]

## Cancers

In recent studies, several advanced nanoparticle-based systems have emerged as effective tools for addressing inflammation and metastasis in cancer therapy [96]. Cationic polyamidoamine (PAMAM) nanoparticles, modified with dodecyl (C12) and diethylethanolamine (DEEA) groups to form G3-C12₅-DEEA₂₀, were engineered to scavenge cell-free nucleic acids. Through enhanced electrostatic interactions provided by their highly cationic surfaces, these nanoparticles were shown to efficiently bind and neutralize both cfDNA and cfRNA. These nanoparticles, with an average size of 140 nm and a + 60-mV zeta potential, demonstrated high paclitaxel loading efficiency (96%) and successfully inhibited Toll-like receptor (TLR3, TLR8, TLR9) activation, reducing the secretion of proinflammatory cytokines TNF-α, IL-1β, and IL-6. In vitro, these nanoparticles reduced the migration and invasion of MDA-MB-231 breast cancer cells (The MDA-MB-231 cell line is derived from metastatic adenocarcinoma which is an advanced stage human breast tumor) by over 50%, and in vivo, they inhibited 73.2% of primary tumor growth, significantly reducing serum cfDNA and cytokine levels. Furthermore, they reduced lung metastasis by more than half compared to free paclitaxel, without showing toxicity in major organs [96].

In another strategy, **c**ationic poly(aspartic acid)–based nanoparticles (cANPs) were developed to inhibit cancer metastasis by scavenging neutrophil extracellular trap DNA (NET-DNA) and disrupting its interaction with the CCDC25 (coiled-coil domain–containing protein 25) receptor on tumor cells. CCDC25 is a cell-surface DNA sensor that recognizes extracellular DNA released from NETs and activates downstream signaling pathways that promote cytoskeletal remodeling, cancer cell migration, and metastatic dissemination. By capturing NET-DNA, cANPs effectively block CCDC25-mediated signal transduction, thereby suppressing metastatic niche formation and tumor spread. This approach highlights the therapeutic potential of nanoparticle-based DNA sequestration strategies for targeting NET-driven metastasis [97]. NET-DNA, the extracellular DNA backbone of NETs released by activated neutrophils and decorated with histones and antimicrobial proteins, can activate CCDC25-mediated signaling pathways that promote tumor cell adhesion, cytoskeletal remodeling, and metastatic dissemination. The cANPs, self-assembled from 100% tertiary-amine–modified poly (aspartic acid), exhibited an average diameter of ~ 120 nm and a zeta potential of + 13.4 mV, providing strong electrostatic affinity for negatively charged NET-DNA and effectively blocking its pro-metastatic interactions. In vitro, cANPs efficiently inhibited NET-DNA binding to CCDC25 and reduced cytoskeleton remodeling, adhesion, and migration of MDA-MB-231 and 4T1 breast cancer cells. In vivo, intravenous administration (10–20 mg/kg) markedly suppressed hepatic and pulmonary metastases in both 4T1 orthotopic and MDA-MB-231 xenograft models, decreasing liver metastatic nodules by over 80%, lowering circulating NET-DNA, and down-regulating pro-inflammatory cytokines (TNF-α, IL-6, IL-1β, IL-17) without inducing organ toxicity [97]. In a broader context, NET-targeting nanotherapeutics are emerging as a powerful class of anti-metastatic interventions, complementing pharmacologic NET inhibitors such as DNase I, PAD4 inhibitors, and anti-inflammatory agents. For example, PEGylated cationic nanoparticles, cationic liposomes, and dendrimeric platforms have been explored for their NET-trapping or DNA-binding capabilities, demonstrating comparable anti-metastatic potential with reduced systemic toxicity. Notably, the cationic poly (aspartic acid) nanoparticles (cANPs) go beyond general DNA scavenging by specifically disrupting the NET-DNA/CCDC25 signaling axis, blocking pro-metastatic interactions and inhibiting metastatic niche formation. With their biodegradability and compatibility with poly (aspartic acid), cANPs exhibit strong translational promise for clinical metastasis control. Future studies that integrate NET-DNA scavenging with immunomodulatory or anti-inflammatory payloads could further enhance therapeutic efficacy against NET-driven cancers and inflammatory pathologies, establishing mechanism-driven, pathway-specific nanoparticle therapy as a frontier in cancer nanomedicine [98].

The broader applications of nanomaterials, including cationic nanoparticles, nanogels, and nanofibers, for the scavenging of cfDNA in tumor metastasis have been highlighted. These materials are designed to capture cfDNA via electrostatic interactions, thereby disrupting cfDNA-mediated activation of immune and tumor-promoting pathways, particularly those involving TLR9 signaling. Their efficacy has been demonstrated in reducing metastatic progression, inflammation, and tissue damage in preclinical cancer models. Moreover, DNase-based systems and nanozymes have been employed to further degrade cfDNA, providing prolonged scavenging effects and enhanced therapeutic outcomes. Among these strategies, cationic nanoparticles with multivalent surface charges have been shown to bind cfDNA most efficiently and accumulate preferentially in tumor-associated or inflamed tissues. Nonetheless, challenges related to nanoparticle stability and potential off-target effects remain, highlighting the need for careful optimization for translational applications [99].

Multifunctional manganese oxide (MnO) nanoparticles were developed to mitigate photothermal therapy (PTT)-induced inflammation during breast cancer treatment, which arises from thermal damage to tumor cells. The nanoparticles were synthesized from tannic acid and manganese acetate, modified with curcumin to confer anti-inflammatory effects, and functionalized with IR780 iodide to enable near-infrared (NIR) light–responsive therapy. They were shown to efficiently scavenge cfDNA and cfRNA, thereby inhibiting TLR activation and NF-κB signaling. In vitro, both MnO and MnO@curcumin nanoparticles were observed to reduce TNF-α and COX-2 mRNA expression, indicating suppression of pro-inflammatory gene transcription. COX-2 (Cyclooxygenase-2) is an enzyme responsible for the production of prostaglandins that drive inflammation and tumor progression, so its downregulation reflects effective mitigation of the inflammatory microenvironment. The nanoparticles also suppressed macrophage migration and inhibited tumor cell invasion. In vivo, these nanoparticles alleviated PTT-induced inflammation, preventing tumor recurrence and metastasis through the scavenging of cfDNA and other danger-associated molecular patterns (DAMPs). Treatment with the nanoparticles resulted in reduced serum cfDNA levels and effectively suppressed lung metastasis, demonstrating superior therapeutic outcomes compared with curcumin alone [100].

These multifunctional nanomaterials (Table 5) provide integrated strategies to scavenge cfDNA, attenuate inflammation, and inhibit metastatic progression, thereby enhancing therapeutic efficacy in cancer treatment while reducing systemic toxicity and off-target effects [101, 102].

**Table 5:** Nanoparticle-mediated cfDNA scavenging for therapeutic applications in cancer

Nanoparticle	Properties	Method	Disease	Quantitative Information	Therapeutic Efficacy (Survival)	Sample	Ref.
Dodecyl and diethylethanolamine–modified polyamidoamine nanoparticle (G3-C12₅-DEEA₂₀)	Average size: 140 nm; Zeta potential: +60 mV; Paclitaxel loading efficiency: 96%	Electrostatic scavenging of cfDNA/cfRNA; inhibition of TLR3/8/9 activation	Breast cancer (MDA-MB-231)	↓ Cytokines (TNF-α, IL-1β, IL-6); ↓ Cell migration/invasion > 50%; ↓ Serum cfDNA and cytokines	↓ Primary tumor growth by 73.2%; ↓ Lung metastasis > 50%; No major organ toxicity	In vitro (MDA-MB-231 cells); In vivo (mouse model)	[96]
ertiary-amine–modified poly (aspartic acid) cationic nanoparticle (cANP)	Size ≈ 120 nm; Zeta potential + 13.4 mV; Strong DNA-binding affinity; High serum stability; Low cytotoxicity (IC₅₀ > 1000 µg/mL)	Electrostatic scavenging of NET-DNA; inhibition of NET-DNA–CCDC25 interaction; suppression of NET formation	Breast and colon cancer metastasis (4T1, MDA-MB-231, HCT116)	↓ NET-DNA binding to CCDC25 by > 50%; ↓ Cytoskeleton remodeling and migration > 80%; ↓ Proinflammatory cytokines (TNF-α, IL-6, IL-1β, IL-17); ↓ Circulating NET-DNA	↓ Hepatic metastasis > 80%; ↓ Lung metastasis; ↑ Survival; No hepatic or renal toxicity	In vitro (MDA-MB-231, 4T1 cells); In vivo (4T1 orthotopic and human xenograft models)	[97]
Cationic nanomaterials including nanogels, nanofibers, and nanozymes (General cationic NMs)	Electrostatic cfDNA binding; DNase and nanozyme activity; Multivalent cationic effects	cfDNA scavenging; inhibition of TLR9 signaling	Autoimmune diseases (RA, SLE) and tumor metastasis	↓ Inflammation and tissue damage; Prolonged cfDNA degradation	Improved therapeutic outcomes; Reduced immune activation	In vitro and in vivo models	[99]
Curcumin- and IR780-modified manganese oxide nanoparticle (MnO@Cur-IR780 NPs)	Synthesized from tannic acid and manganese acetate; Modified with curcumin and IR780; NIR-responsive	cfDNA/cfRNA scavenging; inhibition of TLR and NF-κB signaling	Breast cancer (PTT-induced inflammation)	↓ TNF-α and COX-2 mRNA; ↓ Macrophage migration; ↓ Tumor invasion; ↓ Serum cfDNA	Prevented tumor recurrence and metastasis; Superior to curcumin alone	In vitro (macrophages, tumor cells); In vivo (mouse model)	[100]

## Challenges and future perspectives

The rising global prevalence of diseases such as cancer, rheumatoid arthritis, sepsis, and fetal abnormalities highlights an urgent need for innovative diagnostic and therapeutic strategies. Cancer alone accounts for over 20 million new cases annually, projected to reach 35 million by 2050, while RA affects approximately 18 million individuals worldwide each year. Sepsis impacts 49 million people annually, resulting in 11 million deaths, and fetal abnormalities occur in 2–5% of pregnancies [103–106]. These conditions not only pose severe health challenges but also impose substantial socioeconomic and healthcare burdens. These condition not only pose severe health challenges but also impose substantial socioeconomic and healthcare [107].

CfDNA has emerged as a versatile biomarker, offering dual potential: as a diagnostic indicator and as a contributor to disease-associated inflammation [108]. Its presence in bodily fluids allows for real-time monitoring, personalized therapy guidance, and prediction of disease progression across these diverse pathologies. Despite this promise, several critical challenges limit its clinical translation. The low abundance of cfDNA in circulation, combined with its instability and susceptibility to degradation in complex biological matrices, complicates reliable detection and quantification. Furthermore, the heterogeneous nature of cfDNA, including fragmentation patterns and methylation status, adds layers of complexity for both diagnostics and targeted therapeutics.

To fully exploit cfDNA’s potential, advanced strategies are required that integrate nanotechnology-based delivery and scavenging platforms. Nanomaterials offer opportunities to enhance cfDNA capture, stabilize nucleic acids, and modulate downstream inflammatory responses, thereby bridging gaps between detection, monitoring, and therapy. This review emphasizes these challenges and explores the synergistic application of nanotechnology and cfDNA research, aiming to advance precision medicine approaches for some of the most widespread and impactful diseases.

In diagnostics, functionalized nanomaterials such as Ag⁺, CuS, AuPt nanoparticles, and AuNPs with SPRI have achieved exceptionally low limits of detection (0.3–36 aM) with high recovery rates in patient samples [46, 52, 72]. Additionally, MMNs coated with leukocyte–erythrocyte hybrid membranes can isolate over 90% of FNRBCs with 100% concordance to invasive methods [109]. These advances demonstrate the potential of nanoparticles to enhance cfDNA detection, though further optimization and standardization are needed. An important next step will be the development of reference nanoparticle kits for cfDNA spike–recovery experiments, in which size- and surface-defined nanoparticles are loaded with standardized cfDNA fragments and used as common positive controls across laboratories to harmonize extraction efficiency, limit of detection, and recovery metrics [ref]. Such reference nanoparticle kits would complement existing cfDNA extraction and liquid-biopsy workflows and provide a practical route toward inter-laboratory comparability and regulatory-grade analytical validation of nanoparticle-based cfDNA assays [110].

In therapeutics, cfDNA acts as a pro-inflammatory trigger, activating pathways such as cGAS-STING and MyD88-NF-κB, which exacerbate inflammation and organ injury. cNPs, as well as dual-functional nanoparticles that simultaneously capture cfDNA and release anti-inflammatory drugs like RU.521, have been shown to mitigate these harmful pathways and modulate immune responses. For instance, in CIA mouse models, a NiH system delivering cNPs and RU.521 reduced cfDNA by up to 32%, TNF-α and IL-6 by over 99%, IFN-β by 67%, and increased regulatory T cells by 42% [83]. In sepsis models, TL nanoparticles captured over 94% of cfDNA and improved 7-day survival to 70% [94]. Nonetheless, non-specific interactions and potential off-target effects emphasize the need for selective and safe nanoparticle design.

Artificial intelligence (AI) presents a promising opportunity to optimize detection algorithms, simulate therapeutic responses, and reduce errors and population biases. Integrating AI with nanoparticle platforms could enhance dual-functional systems and advanced diagnostic tools [89, 96].

Ultimately, the future of cfDNA-based nanomedicine depends on overcoming challenges in standardization, clinical validation, and biological specificity. By leveraging AI, dual-functional nanoparticles, and advanced diagnostic platforms, and by expanding applications to underrepresented areas, cfDNA can be transformed into a vital tool for early detection and effective treatment of high-burden diseases.

Truly integrated cfDNA-based theranostic platforms represent the emerging frontier of nanomedicine, in which a single nanoparticle system unifies ultrasensitive detection, quantitative interpretation, and adaptive therapeutic intervention. In this closed-loop paradigm, cfDNA-responsive nanoparticles would first enable sensitive detection and precise quantification of disease-specific cfDNA signatures—including tumor mutation burden, NET-derived DNA, or sepsis-associated cfDNA fragments—using surface-tethered aptamers, molecular beacons, or plasmonic cores, and subsequently initiate readout-guided therapeutic responses only upon confirmation of pathogenic thresholds. Therapeutic activation could involve the release of DNA scavengers, cGAS–STING inhibitors, siRNA, or immunomodulatory agents, thereby minimizing off-target effects and unnecessary immune modulation [111]. Several pioneering proof-of-concept systems already foreshadow this vision, including gold NanoFlare probes that simultaneously detect circulating EMT markers and CTC-derived cfDNA while enabling photothermal therapy from the same nanoparticle scaffold, pH-responsive cationic nanoparticles that scavenge endosomal cfDNA and co-deliver anti-inflammatory cargos with binding-affinity-dependent release kinetics, and magnetic nanoparticle clusters capable of size-selective cfDNA isolation (> 95% recovery of 150–200 bp fragments) suitable for downstream therapeutic activation via aggregation-induced payload deployment [64, 112, 113]. A fully realized cfDNA theranostic nanoparticle would integrate three functional modules: (i) fragment- and mutation-specific cfDNA capture mediated by density-gradient PEG brushes and sequence-selective aptamers; (ii) ratiometric and real-time cfDNA quantification using FRET pairs, SERS, or plasmonic signal amplification to resolve mutant-to-wildtype ratios; and (iii) adaptive therapeutic release governed by disulfide- or proteinase-cleavable cages that liberate DNase mimetics, pathway inhibitors, or immunoregulatory agents once cfDNA concentrations exceed clinically relevant thresholds (e.g., > 10 ng/mL). Quantitative diagnostic outputs could further trigger DNAzyme cascades or CRISPR-gated amplification circuits to fine-tune therapeutic dosing in dynamic disease states such as sepsis, metastatic cancer, or autoimmune flares [114]. By transforming circulating cfDNA from a passive biomarker into an intelligent molecular decision node, such integrated theranostic platforms would fundamentally redefine precision nanomedicine, and their clinical realization will depend on parallel advances in standardized reference nanoparticle kits, AI-guided surface and payload optimization, and clearly defined translational and regulatory pathways [111].

## Supplementary Information

Supplementary Material 1

## Data Availability

No datasets were generated or analysed during the current study.

## Abbreviations

5hmC: 5-hydroxymethylcytosine
aM: attomolar
AgNPs: Silver nanoparticles
Au@MNPs: Gold-coated magnetic nanoparticles
AuNPs: Gold nanoparticles
AuPt: Gold–platinum alloy nanoparticles
cfDNA: Cell-free DNA
cffDNA: Cell-free fetal DNA
cGAS: Cyclic GMP–AMP synthase
COFs: Covalent organic frameworks
CRC: Colorectal cancer
ctDNA: Circulating tumor DNA
CuS: Copper sulfide
DAMP: Damage-associated molecular pattern
DPV: Differential pulse voltammetry
DTSP: Dithiobis(succinimidyl propionate)
EDC/NHS: 1-Ethyl-3-(3-dimethylaminopropyl)carbodiimide / N-hydroxysuccinimide
EGFR: Epidermal growth factor receptor
fM: femtomolar
Fe₃O₄: Iron(II, III) oxide (magnetite)
HAC-AuPt: High-active carbon-supported AuPt alloy nanoparticles
HCC: Hepatocellular carcinoma
H₂O₂: Hydrogen peroxide
KRAS: Kirsten rat sarcoma viral oncogene homolog
LB: Leucomethylene blue
LOD: Limit of detection
LSPR: Localized surface plasmon resonance
MB: Methylene blue
mAC + DC: Magnetic-activated capture and digital counting
MPNPs: Magneto-plasmonic nanoparticles
MOFs: Metal–organic frameworks
MNPs: Magnetic nanoparticles
NGS: Next-generation sequencing
NIPT: Non-invasive prenatal testing
NPs: Nanoparticles
NSCLC: Non-small cell lung cancer
PCR: Polymerase chain reaction
PIK3CA: Phosphatidylinositol-4,5-bisphosphate 3-kinase catalytic subunit alpha
PNA: Peptide nucleic acid
pM: picomolar
qPCR: Quantitative polymerase chain reaction
QDs: Quantum dots
RA: Rheumatoid arthritis
RBCs: Red blood cells
RSD: Relative standard deviation
SERS: Surface-enhanced Raman spectroscopy
SNVs: Single-nucleotide variants
SPRI: Surface plasmon resonance imaging
STING: Stimulator of interferon genes
TACN: Tetra-azacyclononane (used in hmC TACN assay)
TKI: Tyrosine kinase inhibitor
TLR9: Toll-like receptor 9
UCNPs: Upconversion nanoparticles
VAF: Variant allele frequency
