# Supplementary material for: Cell-free DNA in precision medicine: overcoming biological barriers through integrated nanoparticle platforms for simultaneous diagnosis and therapy
Source: J Nanobiotechnology. 2026 Feb 27;24:308. doi: 10.1186/s12951-026-04220-9 (PMC13049810; doi:10.1186/s12951-026-04220-9)
Supplement: Supplementary file 1 — Supplementary Material 1 [file 12951_2026_4220_MOESM1_ESM.docx]

**Supplementary Table S1.** Nanoparticle-based ctDNA detection platforms

| Nanoparticle Type | Name | Factor Detected | Cancer Type | Limit of Detection (LOD) | Linear Range | Key Factors | References |
| --- | --- | --- | --- | --- | --- | --- | --- |
| Gold Nanoparticles (AuNPs) | AuNPs with COFs | *EGFR* L858R | NSCLC | 0.35 fM | 1 fM-100 nM | LSPR, high surface area, electrochemiluminescence, 99.4% stability (2 weeks), recovery: 97.4-106% | [39] |
|  | AuNPs with COF@Au-PEI | *EGFR* T790M | NSCLC | 0.6 pM | 10⁻¹²-10⁻⁷ M | LSPR, conductivity, electrochemical, 1.4-5.6% stability (21 days), accuracy: >95.5% | [51] |
|  | AuNPs (65 nm) | *EGFR* L858R | NSCLC | 0.1 nM | - | LSPR shift, UV-Vis/DLS, ΔR=0.6, single-nucleotide polymorphism detection | [69] |
|  | AuNPs (~10 nm) | *PIK3CA* mutations | Breast, Colorectal, Brain, Endometrial | - | - | Increased surface area, chronoamperometry, discrimination factor: 2.7 | [70] |
|  | AuNPs with ZnO NRs | 5hmC | HCC | 0.236 pmol/µg | - | LSPR, Conductivity, Electrochemical (CV), 2–3× lower current, P < 0.0001 | [71] |
|  | AuNPs (40 nm) | miR-31 (exosomal) | Colon | - | - | Fluorescence quenching, CD81 conjugation, 97% miR-31 decrease, P = 1.7 × 10⁻⁵ | [72] |
|  | AuNPs (13 nm) | *EGFR* L858R | NSCLC | 0.22 fM | - | Fluorescence quenching, FEN1-DNA walker, 100% NGS concordance | [73] |
|  | AuNPs with Poly-L-lysine (PLL) | *KRAS* (p.G12D, p.G13D) | CRC | ~2.5 aM | - | SPR enhancement, SPR imaging, signal ratio: 1.20 vs. 0.86, P = 7.15 × 10⁻⁵ | [45] |
|  | AuNPs (20 nm) | *EGFR* exon 19 deletion | NSCLC | 38.5 aM | - | LSPR, UV-vis absorbance, 0.1% selectivity | [42] |
|  | AuNPs (20, 60 nm) | *BRCA1* cfDNA | Breast | 0.34 fM | 1 fM–100 pM | LSPR, MEF, fluorescence quenching, <30 min reaction time | [43] |
|  | AuNPs (nanobipyramids, 25×70 nm) | *KRAS* G12D | Cancer | ~1 ng/mL | 0–100 ng/mL | LSPR, RI sensitivity, 5-fold mutant vs. wild-type selectivity | [74] |
|  | AuNPs (LSPR biosensor) | cfDNA | Ovarian, Rectal | 14.0 pM | - | LSPR, HCR, 88.54–112.80% recovery, RSD 2.66–4.20% | [75] |
| Silver-Nanoparticle (AgNPs) | Ag@N,O-C BLHS | *PIK3CA* E545K | Breast | 10 aM | 10⁻¹⁶–2×10⁻¹⁰ M | High surface area, electrochemical, 97.5% stability (14 days), median: 36.46 fM | [52] |
| Gold-Silver Nanoparticles  (Au@AgNPs) | Gold-core Silver-shell NPs (Au@AgNMs) | *MUC1* (CTCs) | Breast | 5 cells/mL | 10–10⁴ cells/mL | Plasmonic hotspots, SERS/fluorescence, DNA walkers, recovery: 90–126% | [76] |
|  | AuNPs, AgNPs | SHOX2, RASSF1A methylation | NSCLC | m-SHOX2: 0.52 pM  m-RASSF1A: 0.66 pM | Varies | SERS enhancement, high specificity | [68] |
| Copper Nanoparticles | CuS NPs with DNA@Ag+ | *EGFR* L858R | Lung | 0.3 aM | 1 aM–1 fM | Chemical reactivity, electrochemical, DNA nanomachine, 96% stability (12 hours), AUC: 0.97 | [46] |
|  | CuNPs | 5hmC | Breast, Lung, Liver, Bladder | 64 pM | 0.097–0.81% | Fluorescence (Ex: 345 nm, Em: 627 nm), hmC-TACN method | [55] |
| Zinc Oxide Nanostructures | ZnO Nanowires | *IDH1* R132H | Glioma | - | - | High binding affinity, capture/PCR, ~90% capture, 0.11 ng/µL cfDNA | [47] |
| Cadmium Sulfide Quantum Dots (CdS QDs) | CdS QDs with AuNPs | *EGFR* T790M | NSCLC | 3.5 aM | 5 aM–100 fM | Electrochemiluminescence, LOD (plasma): 91 aM | [54] |
| Superparamagnetic Nanoparticles | Superparamagnetic Beads | ctDNA | General (Stage I/II) | - | - | Superparamagnetism, silica shell, 5.7 ng/10 mL, 65.57% sensitivity, 95.38% specificity | [27] |
|  | Fe₃O₄-CA | Methylated E-cadherin | Breast, Gastric, Prostate, Thyroid, Colorectal | 9×10⁻⁵ ng/mL | - | Superparamagnetism (55.72 emu·g⁻¹), electrochemical, 97–100.66% recovery | [76] |
|  | Fe₃O₄ NPs with UCNPs | PIK3CA | Cancer | 1.6 aM | 100 aM–1 nM | Superparamagnetism, ESDR, single-base mismatch discrimination | [77] |
|  | Magnetic Zirconium-Organic Framework (MZMOF) with Fe₃O₄ | DNA (129 bp, 3134 bp) | Infectious Diseases, cfDNA | - | - | Magnetism, 90.4–95.1% recovery, IFAST chip <10 min | [78] |
| Gold-Coated Magnetic Nanoparticles | Au@MNPs | NSCLC ctDNA | NSCLC | 5 fM | 200 aM–20 nM | Magnetic dispersion, electrochemical activity, CV/DPV, 20-minute assay | [28] |
| Gold-Platinum Nanoparticles | AuPt with HAC | ctDNA | Cancer | 3.6 × 10⁻¹⁷ M | 10⁻⁸–10⁻¹⁶ M | Catalytic, conductive, electrochemical, 92.6–105.1% recovery | [49] |
| Upconversion Nanoparticles (UCNPs) | NaYF₄ (Yb³⁺/Tm³⁺) with AuNCs | ctDNA | Cancer | 6.30 pM | 5 pM–1000 pM | Upconversion luminescence, FRET, high specificity | [35] |
|  | UCNPs with Fe₃O₄ NPs | PIK3CA | Cancer | 1.6 aM | 100 aM–1 nM | Upconversion fluorescence, ESDR, single-base mismatch discrimination | [77] |
| Quantum Dots (QDs) | QDs (525, 625, 705 nm) | Trimethylated H3, methylated cfDNA | Cancer, Diabetes | 0.001 ng | - | Size-dependent luminescence, multiplex detection | [43] |
| Metal-Organic Frameworks (MOFs) | ZIF-8 with AuNPs | miRNA-21 (cfDNA proxy) | Cancer | 29 pM | 0.1 nM–10 µM | Porosity, electrochemical activity, RSD 5.1% | [67] |
| Cationic Nanogels (cNGs) | cNGs | cfDNA | Chronic Wounds | - | - | Electrostatic interaction, Ka ~4.13 × 10⁵ M⁻¹, 96% sequestration | [79] |
| Hybrid Nanostructures | AuNPs, AgNPs, Carbon NPs | AFP-L3 | HCC | aM range | Varies | Light absorption, photoelectrochemical, high sensitivity | [44] |
|  | AuNPs, QDs, UCNPs | PIK3CA, KRAS, EGFR | Multi-cancer | aM–fM | Varies | Fluorescence, LSPR, matches qRT-PCR | [80] |

**Table S 2.** Summary of cNPs for Suppression of cfDNA-Mediated Inflammation in RA


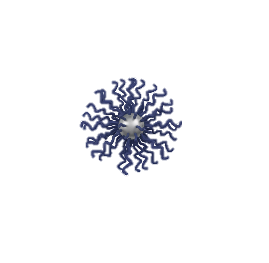

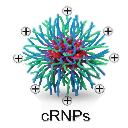

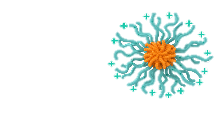

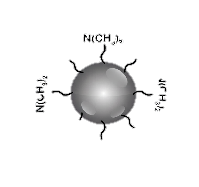

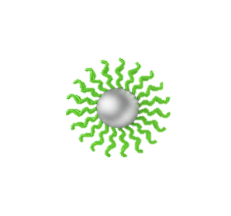

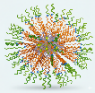


| **Nanoparticles** | **cRNPs in NiH** | **SiNP@PDMA** | **DP-B(H)** | **cNP2** | **MTX@cNP-pp-PEG** | **PLGA-b-PDMA** |
| --- | --- | --- | --- | --- | --- | --- |
| **Core Material** | RNA-based nanoparticle | Silica | Polydopamine | Polymer-based | PLGA | PLGA |
| **Shell / Coating** | Hydrogel-embedded | PDMA cationic brush | Not separate; core-shell is same (biodegradable) | Dual hydroxyl shell on cationic PDMA | PDMA cationic shell + PEGylation | PDMA cationic  shell |
| **Surface Functionalization** | Amphiphilic miktoarm star polymer; cationic for cfDNA capture | Tunable degree of polymerization | Positive surface charge | Dual hydroxyl shell on cationic PDMA | PDMA cationic shell + PEGylation | PDMA cationic  shell |
| **Drug Loading** | RU.521 in separate nanoparticles (~63 nm) | None | None | None | Methotrexate (93.7% encapsulation) | None |
| **Mechanism** | Dual system: cfDNA scavenging + delivery of cGAS inhibitor | cfDNA scavenger; size-dependent accumulation and retention; enhanced electrostatic interactions | cfDNA scavenger; biodegradable; reduces inflammation in CIA rats | Strong cfDNA binding, inhibits TLR9, improved circulation and joint targeting | cfDNA scavenger + drug delivery; stimulus-responsive (MMP2) for controlled release | Blocks cfDNA-TLR9 activation; smaller size; some toxicity at high doses |
| **References** | [83] | [84] | [85] | [86] | [87] | [29] |
